# Molecular Strategies of the *Caenorhabditis elegans* Dauer Larva to Survive Extreme Desiccation

**DOI:** 10.1371/journal.pone.0082473

**Published:** 2013-12-04

**Authors:** Cihan Erkut, Andrej Vasilj, Sebastian Boland, Bianca Habermann, Andrej Shevchenko, Teymuras V. Kurzchalia

**Affiliations:** 1 Max Planck Institute of Molecular Cell Biology and Genetics, Dresden, Germany; 2 Max Planck Institute of Biochemistry, Martinsried, Germany; Centre National de la Recherche Scientique & University of Nice Sophia-Antipolis, France

## Abstract

Massive water loss is a serious challenge for terrestrial animals, which usually has fatal consequences. However, some organisms have developed means to survive this stress by entering an ametabolic state called anhydrobiosis. The molecular and cellular mechanisms underlying this phenomenon are poorly understood. We recently showed that *Caenorhabditis elegans* dauer larva, an arrested stage specialized for survival in adverse conditions, is resistant to severe desiccation. However, this requires a preconditioning step at a mild desiccative environment to prepare the organism for harsher desiccation conditions. A systems approach was used to identify factors that are activated during this preconditioning. Using microarray analysis, proteomics, and bioinformatics, genes, proteins, and biochemical pathways that are upregulated during this process were identified. These pathways were validated via reverse genetics by testing the desiccation tolerances of mutants. These data show that the desiccation response is activated by hygrosensation (sensing the desiccative environment) via head neurons. This leads to elimination of reactive oxygen species and xenobiotics, expression of heat shock and intrinsically disordered proteins, polyamine utilization, and induction of fatty acid desaturation pathway. Remarkably, this response is specific and involves a small number of functional pathways, which represent the generic toolkit for anhydrobiosis in plants and animals.

## Introduction

Terrestrial organisms encounter limited water supplies seasonally or permanently. To survive, many organisms have evolved strategies to respond to this challenge. Anhydrobiosis (life without water) is a widespread phenomenon observed in all taxa of life [1-8]. Some anhydrobiotic organisms (anhydrobiotes) can live without water for centuries or longer, showing no measurable metabolism (ametabolism) [9,10]. Upon rehydration, they quickly exit this dormancy and recover metabolic activity by poorly understood mechanisms.

Anhydrobiosis has probably been best studied in resurrection plants, which can survive losing more than 95% of the free water in their vegetative tissues. These plants initially react to desiccation stress by abscisic acid-mediated expression of stress-resistance genes such as aldehyde dehydrogenases, heat shock proteins (HSPs), and late embryogenesis abundant (LEA) proteins [11]. This initial response is followed by the inhibition of photosynthesis, which is thought to prevent reactive oxygen species (ROS) production. Additionally, accumulation of sugars (mainly sucrose [12] but also trehalose [13]) and the expression of HSPs and LEA proteins are suggested to protect membranes and proteins in the desiccated state [11,14].

In contrast to plants, the molecular strategies underlying anhydrobiosis in animals are poorly understood, although some of the aforementioned pathways in plants were also discovered in animals [1,15-19]. This gap in knowledge is mostly due to the lack of an established genetic animal model. We recently showed that one of the best-studied model organisms in biology, the nematode *C. elegans*, is an anhydrobiote [19,20]. The non-reproductive “dauer” larvae [21] of these worms can survive even after losing 98% of their body water. However, this occurs only if worms are prepared/preconditioned, i.e., kept at conditions of mildly reduced humidity (98% relative humidity [RH]) for several days before they are exposed to harsh desiccation. During preconditioning of dauer larvae, the level of the disaccharide trehalose increases 4-fold, which appears to maintain the native packing of membrane lipids in the desiccated state [19,20]. Thus, dauer larva can detect a decrease in humidity and react to it by activating specific biochemical pathways. Trehalose is apparently not the only protective factor: trehalose-deficient mutants can lose substantial amounts of water and remain viable at moderate humidity levels (80% RH), although their desiccation tolerance falls dramatically as humidity decreases. Furthermore, bdelloid rotifers can undergo anhydrobiosis without producing any trehalose [22] and some tardigrades have very low levels of trehalose, which are not increased upon desiccation [23]. Therefore, a systematic characterization of biochemical pathways is required to understand the molecular mechanisms underlying anhydrobiosis.

Preparation for desiccation should begin with sensing the decrease in humidity. However, it is not known how worms sense these changes or whether cells behave autonomously or they are centrally regulated. The ability of an animal to sense a change in ambient humidity, also known as hygrosensation, was first studied at the molecular level in fruit flies [24] and was recently associated with the transient receptor potential (TRP) channels Nanchung, Inactive, and Water witch that are expressed in neurons [25]. Additionally, a ground beetle was shown to have hygroreceptors, although the proteins that constitute these receptors have not been characterized [26]. As for many organisms, the cellular and molecular mechanisms underlying hygrosensation in *C. elegans* remain unknown.

In this study, we applied a systems approach to explore the molecular strategies of desiccation tolerance in *C. elegans* and in particular, how the worm reacts to a mild desiccation stress (preconditioning). For this, changes in the transcriptome and proteome of the dauer larva during preconditioning were surveyed. Using more than 30 mutant strains or RNA interference (RNAi)-induced knockdowns, we investigated how desiccation tolerance is affected when these pathways are perturbed. Our results indicate that desiccation tolerance of *C. elegans* depends on a small number of functional pathways that are conserved among plants and animals, which can be a generic toolkit for anhydrobiosis.

## Results

### Concept and Experimental Design

We used a systems approach to elucidate the genes and proteins that are induced or activated in response to desiccation stress. The outcome of this analysis was used to define candidate anhydrobiotic pathways, which were validated by a standardized desiccation tolerance assay on mutant worms or worms treated with RNAi.

First, the dauer larva should have a mechanism that senses a decrease in ambient humidity, namely hygrosensation. The signal of a desiccative environment, regardless of how it is detected, should be transformed into a response that is manifested at all levels of gene expression: transcriptional, translational, and post-translational. Figure S1A shows possible scenarios of this response in the *C. elegans* dauer larva. Desiccation tolerance relevant (DTR) mRNAs or proteins could be produced during dauer formation. Upon preconditioning, these DTR mRNAs can be activated and additional desiccation-induced DTR mRNAs can be synthesized *de novo*. Both types of DTR mRNAs can lead to *de novo* synthesis of DTR proteins. Hygrosensation can also induce post-translational modifications (e.g., phosphorylation or glycosylation) of these proteins. DTR proteins will then execute the functions required for anhydrobiosis.

To explore these scenarios, we analyzed the differential expression of genes at the mRNA level and the corresponding changes at the proteome level, as well as the post-translational modifications of proteins. For this purpose, we used microarray analysis, gel electrophoresis liquid chromatography tandem mass spectrometry (geLC-MS/MS), and 2-dimensional difference gel electrophoresis (2D-DIGE) (Figure S1B). A temperature-sensitive dauer-constitutive strain of *C. elegans*, *daf-2*(*e1370*), was grown in liquid culture at 25 °C until complete dauer formation [19]. One aliquot of these worms was immediately frozen before any desiccation, and was thus treated as a non-desiccated control. The rest were subjected to mild desiccation at 98% RH for 1 day or 4 days at 25 °C (preconditioning). Dauer larvae were collected and total RNA and protein were extracted for transcriptome and proteome analysis, respectively (Figure S1B, upper part). To test the reversibility of changes in the proteome during preconditioning, an aliquot of the 4-day-desiccated worms was kept in water for 24 h to rehydrate. Subsequently, proteins were also extracted from this sample.

Various bioinformatics tools were used to identify candidate genes and pathways that were differentially expressed during preconditioning, and thus presumably have a function in the desiccation response. Finally, to assign functions to these pathways, we tested the desiccation tolerance of mutant worms deficient in genes of the pathway or used an RNAi knockdown approach (Figure S1B, lower part).

### Microarray Analysis of Genes That Are Induced Upon Desiccation

**Figure 1:**
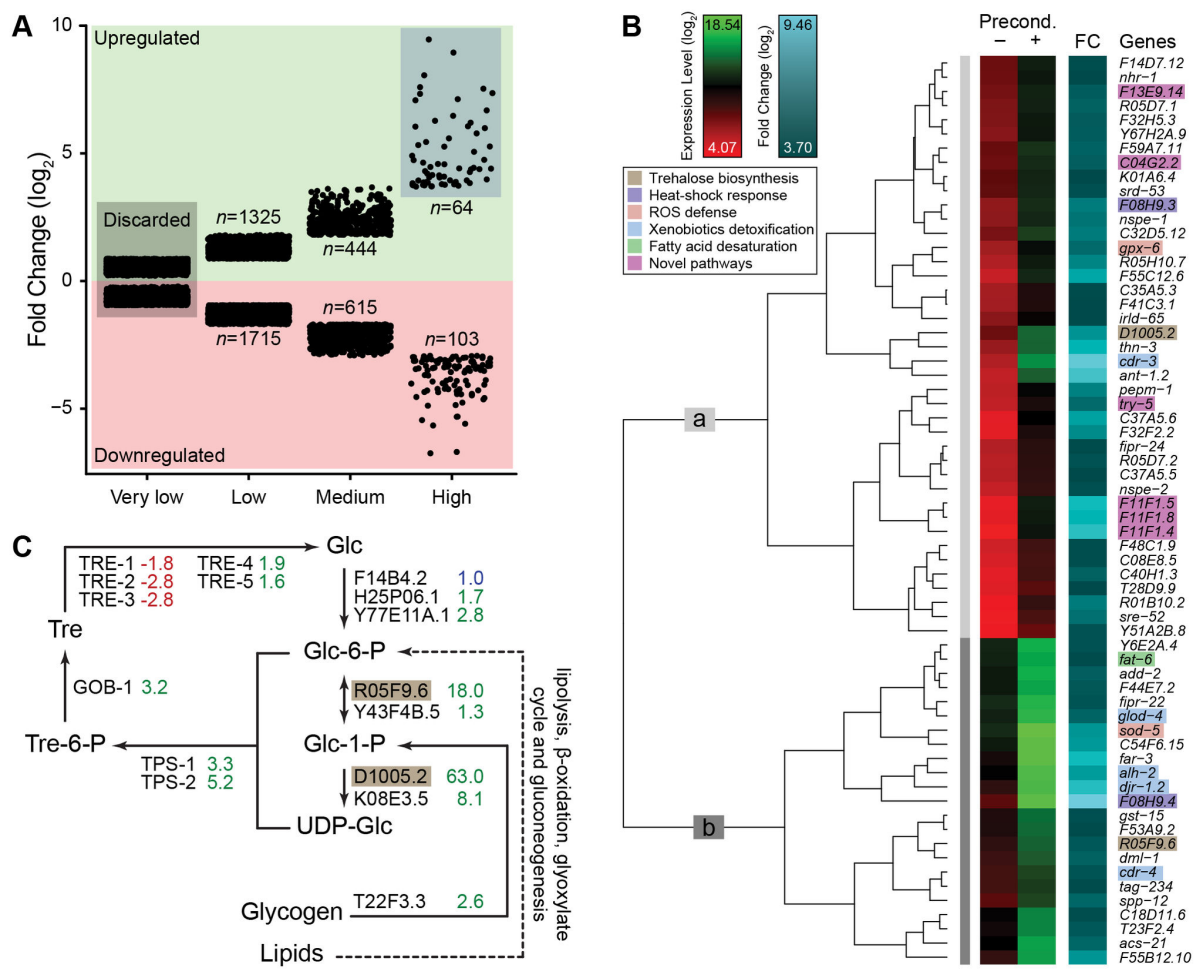
Analysis of desiccation-induced DTR mRNAs. (A) Fold change clustering of upregulated (green area) and downregulated (pink area) genes. The very low FCC was discarded because the differential expression was too low. The number of genes (*n*) in each group is shown. (B) Hierarchical clustering of the 64 highly upregulated genes (blue region in panel A). Red/green column shows log_2_ expression levels before (–) and after (+) preconditioning, cyan column shows log_2_ fold changes for the genes indicated on the right. Color codes for the heat map are shown top left. Main branches of the dendrogram are labeled ‘a’ (light gray) and ‘b’ (dark gray, see the text for details). Highlighted genes reside in essential anhydrobiotic pathways shown in the legend with unique color codes, which are consistent in the following figures. (C) The trehalose biosynthesis pathway is upregulated in *C*. *elegans* upon desiccation stress. Enzymes that catalyze each reaction are shown with the corresponding differential expression values of their transcripts upon preconditioning. These fold changes are shown in green, red, and blue for upregulation, downregulation, and no change, respectively. Glucose-6-phosphate synthesis from lipids involves several steps; therefore, a dashed arrow is used. Highlighted genes are found in the high FCC (panel B). Glc, Glucose; Glc-6-P, glucose-6-phosphate; Glc-1-P, glucose-1-phosphate; UDP-Glc, UDP-glucose; Tre-6-P, trehalose-6-phosphate; Tre, trehalose.

Using microarray technology, we surveyed the differential expression of 20,058 protein-coding genes of *C. elegans* in the dauer larva before and after preconditioning. During the first day of exposure to decreased RH, 4,764 (23.7%) of these genes were significantly upregulated and 4,791 (23.9%) were significantly downregulated (p < 0.001). However, the majority of these genes had very small differential expression levels (fold changes, FCs), and were therefore regarded as being biologically non-relevant. To filter these genes out without using arbitrary FC cutoffs, we used k-means clustering on FC values. This is one of the simplest unsupervised machine learning algorithms that divides a dataset into a predefined number of subsets by optimizing the distance between means. Using this algorithm, we grouped all significantly upregulated and downregulated genes into four sets: very low, low, medium, and high FC clusters (FCCs) (Figure 1A). The very low FCC revealed by k-means clustering (less than 1.82-fold upregulation and 1.92-fold downregulation) was excluded from further analyses. Finally, 1,833 (9.1%) upregulated and 2,433 (12.1%) downregulated genes remained (Dataset S1). These genes were considered to be statistically significant and biologically relevant.

Most interesting for further analysis were two FCCs: medium (444 genes with 3.5–13 FC) and high (64 genes with at least 13 FC) (Figure 1A). The top 10 genes were upregulated by more than 100-fold upon preconditioning. We considered the genes in the high FCC as major candidates to have specific functions in desiccation tolerance. Therefore, they were expected to be expressed at relatively low levels before preconditioning. We tested this hypothesis by clustering these genes according to their expression levels in non-preconditioned and preconditioned samples (Figure 1B). Here, we took advantage of the one-color microarray hybridization technology in which the signal intensity of a probe correlates with the abundance of its target mRNA molecule, i.e., its expression level [27]. Hierarchical clustering of the control and preconditioned expression levels of high FCC genes resulted in two patterns: about half of them were expressed at low levels before preconditioning and their transcript levels increased enormously upon desiccation stress (Figure 1B, branch a). The remaining genes were expressed at a medium level before preconditioning and were induced further upon desiccation, often eventually being expressed at the highest levels (Figure 1B, branch b). Thus, genes of the first pattern appear to be specific for the desiccation response, whereas genes of the second pattern might also be involved in the regular functioning of the dauer and be required prior to desiccation. Alternatively, they might be necessary to provide a basal level of desiccation tolerance to the dauer, which is increased upon desiccation stress by the activation of DTR genes and proteins.

One possible way to analyze the desiccation stress response would be to investigate the roles of individual genes identified by microarray analysis. However, many *C. elegans* genes are functionally highly redundant [28], which would make such analysis extremely difficult, if not impossible. Therefore, we first assigned the identified candidates to biochemical and genetic pathways and then investigated the involvement of the whole pathway in desiccation tolerance. As a proof of principle, we tested whether the strong increase of trehalose during preconditioning, which we observed in our previous study [19], was reflected in the current microarray data. The biosynthetic pathway of trehalose and the genes involved in this pathway are shown in Figure 1C. Worms synthesize trehalose in a two-step reaction from glucose-6-phosphate and UDP-glucose. The first step is catalyzed by trehalose-6-phosphate synthase, which is encoded by two paralogous genes in *C. elegans*: *tps-1* and *tps-2* [29]. Trehalose-6-phosphate is then dephosphorylated by trehalose-6-phosphate phosphatase, yielding trehalose [30]. Several enzymes catalyze the production or conversion of the substrates required for TPS-1 and TPS-2 (note the genetic redundancy of the enzymes in the pathway). As seen in Figure 1C, all the genes encoding the enzymes involved in trehalose production were upregulated upon preconditioning with the exception of an isoform of hexokinase, F14B4.2, which remained unchanged. Most importantly, the genes encoding two enzymes (phosphoglucomutase R05F9.6 and UDP-glucose pyrophosphorylase D1005.2) were found in the high FC cluster (highlighted in brown in panels B and C). In addition, three of the five trehalose hydrolyzing enzyme (trehalase) genes were downregulated. These data clearly show that microarray data can be used to predict the pathways involved in the desiccation response in worms.

Next, we performed functional annotation clustering of differentially expressed genes based on their Gene Ontology and conserved protein domain annotations (Dataset S2, summarized in Table S1) [31,32]. These clustering results suggested that activation of many enzymatic processes participates in lipid and sugar metabolism. Especially, upregulation of lipid-binding protein, lipase, fatty acid (FA) desaturase (FAT), and lipid glycosylation enzyme genes suggests that lipidome remodeling can be intensive during preconditioning. At the same time, transcriptional activation of genes involved in mitochondrial substrate/solute carrier activity could be an indication of elevated energy production during preconditioning, at least in the early stages. The data also indicated strong activation of the ROS defense pathway. Additionally, many enzymes with cytochrome P450 and glutathione S-transferase (GST) activity were enriched among the upregulated genes. These enzymes not only accept endogenous substrates but also contribute to degradation of xenobiotics. Thus, detoxification may be another general strategy of desiccation tolerance.

Overall, it seems that during entry into anhydrobiosis, worms reduce or cease building new morphological structures (e.g., cuticle) while preserving the existing ones. This is evident from the downregulation of cuticle and extracellular matrix proteins, lipid glycosylation enzymes, and proteases, as well as lysozymes that break down peptidoglycans. Amino acid metabolism and transport are also reduced, which might be an indication of the onset of metabolic depression in conjunction with decreased lipase activity (Table S1).

### The *C. elegans* Dauer Larva Responds to Desiccation at the Translational and Post-translational Levels

To survey the desiccation-induced changes at the proteome level, we used two approaches: a label-free protein quantification method geLC-MS/MS [33] and a minimal-labeling method 2D-DIGE. geLC-MS/MS detected 1,058 proteins expressed in the *C. elegans* dauer. However, the relative abundance of the majority of these proteins did not significantly change during desiccation. After 1 day of desiccation stress, the abundance of only 17 (1.6%) proteins was significantly increased. On the fourth day, the levels of 12 of these 17 proteins were further increased or whereas 5 remained unchanged. Additionally, the abundance of 22 other proteins increased and the abundance of nine proteins decreased, resulting in a total of 39 (3.7%) upregulated and 9 (0.9%) downregulated proteins (Dataset S3). These results suggest that desiccation-induced protein expression is very specific. It also indicates that desiccation mainly induces protein biosynthesis rather than protein degradation, as very few downregulated proteins were detected.

Proteins whose abundance was most strongly altered during preconditioning were mainly ROS defense and detoxification enzymes, FA- and retinol-binding proteins, HSPs, and intrinsically disordered proteins (IDPs), as well as transthyretin-like proteins. These classes correlated quite well with the data obtained from the microarray analysis of DTR mRNAs that are upregulated during preconditioning. The first 22 proteins with at least 2 FC, or their paralogs, were also found in the medium or high FCCs of the microarray survey. The only exception was the uncharacterized protein F57H12.6, which was found exclusively by the geLC-MS/MS approach. Its expression remains unchanged according to the microarray data.

**Figure 2:**
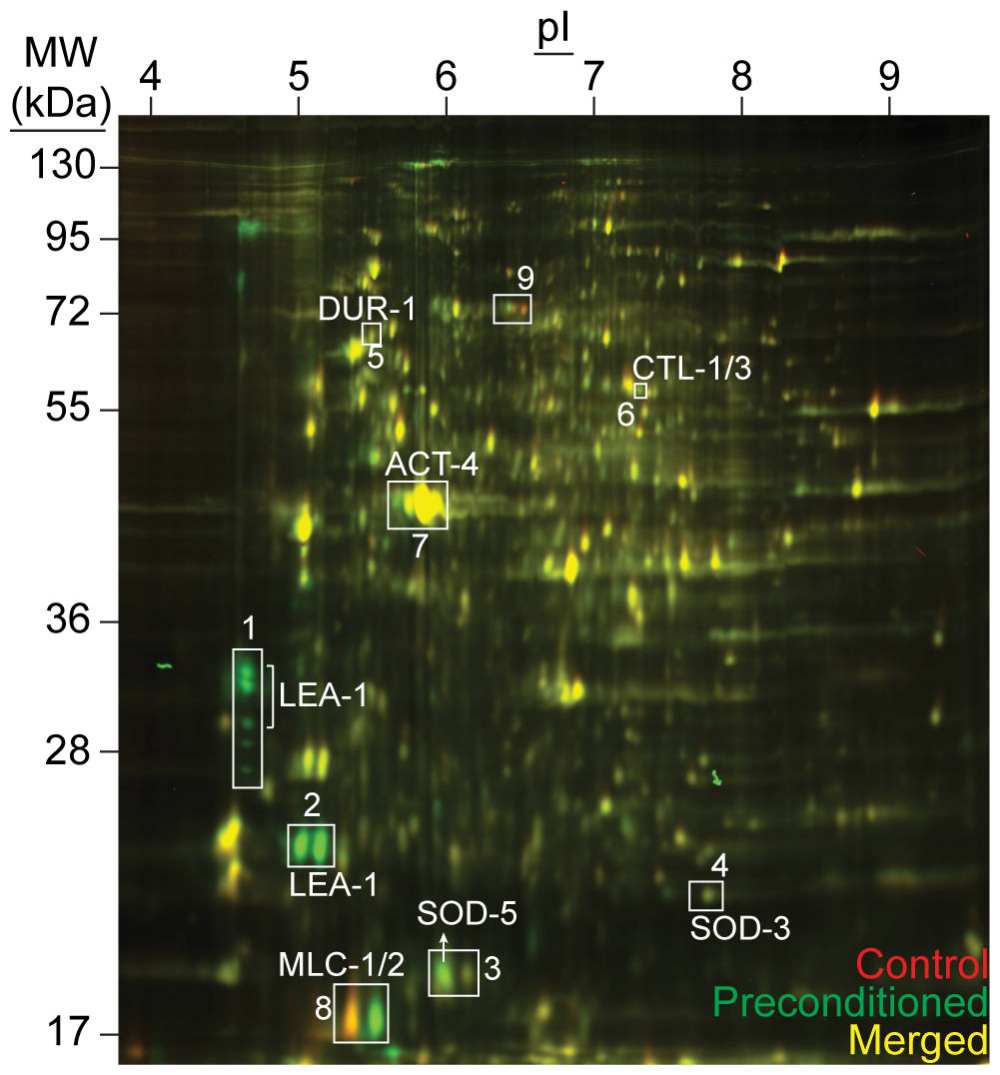
Comparison of proteomes upon desiccation. Overlay of false-colored 2D-DIGE images comparing dauer proteomes before (red) and after (green) preconditioning at 98% RH for 4 days. Yellow spots indicate proteins that do not change distinctively. The major proteins identified in these spots are annotated. The numbered regions are shown in higher magnification in Figures S2C–E. For MLC-1/2 (region 8), the red and green spots were identified as phosphorylated and dephosphorylated proteins, respectively. Region 9 possibly shows another post-translational modification.

Another powerful, unbiased tool to identify differential expression at the protein level is 2D-DIGE. In comparison to geLC-MS/MS, this method has notable benefits in revealing changes in the patterns of post-translational modifications of proteins. 2D-DIGE of protein extracts from non-desiccated and 4-day-desiccated dauer larvae is shown in Figure 2 (red and green spots, respectively). The abundance of the majority of proteins did not change during desiccation (yellow spots), which corroborates the microarray and geLC-MS/MS findings. Remarkable *de novo* synthesis of several isoforms of LEA-1, an intrinsically disordered protein, was noted in the desiccated samples (Figures 2, S2B, S2C). The implications of this are discussed below.

Well known as a stress-resistant stage, the dauer larva is remarkably different from its reproductive stage counterpart L3 larva. These differences might also contribute to the desiccation tolerance. We addressed this hypothesis by comparing the proteomes of L3 and dauer larvae using 2D-DIGE (Figure S2A). Clearly, the two stages differ dramatically at the protein level. While some proteins such as SOD-3 are also expressed in L3 larvae, although at lower levels, others such as DUR-1, CTL-1, and CTL-3 are entirely dauer-specific (Figures S2A, S2D). This indicates that the transition to dauer might confer partial desiccation tolerance ability, and that full desiccation tolerance is acquired upon desiccation stress.

In addition to protein expression, desiccation also induces post-translational modifications to existing proteins. Phosphorylation of actin and dephosphorylation of myosin light chain proteins were detected upon desiccation (Figures 2, S2E).

Interestingly, upon rehydration, many DTR proteins were downregulated to their basal levels although the levels of some proteins remained elevated (Figures S2B, S2C). Desiccation-induced post-translational modifications were also reversed in many cases upon rehydration (Figures S2B, S2E).

Data obtained by geLC-MS/MS and 2D-DIGE complement those of the microarray survey and thus were incorporated into efforts to identify the pathways responsible for desiccation tolerance.

### Major Biochemical Pathways Underlying Desiccation Tolerance

Unbiased bioinformatics examination of the transcriptome and proteome data led to the identification of candidate pathways that were induced by desiccation. Nevertheless, subsequent biological validation was essential to associate these pathways with anhydrobiosis. Therefore, we tested selected genes in some of these pathways for their involvement in desiccation tolerance (Table 1). Dauer larvae carrying mutations in the listed genes were first preconditioned at 98% RH for 4 days. Then they were either kept at 98% RH for another day or subjected to a harsher desiccation at 60% RH, which cannot be tolerated in the absence of essential DTR genes such as *tps-1* and *tps-2* [19]. Finally, the percentage of survivors was calculated by counting the worms. When mutant strains were unavailable, we silenced the candidate gene by RNAi on *daf-2* background and performed the same desiccation assay. In these experiments, *daf-2* eggs were grown on RNAi feeding plates at 25 °C for one generation until they form dauer larvae. Every mutant or RNAi knockdown was tested at least twice. In every desiccation experiment, wild type (N2) or RNAi non-treated *daf-2* worms were used as the control.

**Table 1:** Selected pathways induced by desiccation stress and their genetic components that were functionally investigated in this study.

**Category**	**Gene**	**Description**	**Fold Change** ^a^
Reactive oxygen species defense	*sod-5*	Superoxide dismutase	66 (90)
	*gpx-6*	Glutathione peroxidase	27
	*sod-3*	Superoxide dismutase	7
	*gpx-2*	Glutathione peroxidase	6.1
	*sod-1*	Superoxide dismutase	2.8 (1.6)
	*gpx-7*	Glutathione peroxidase	1.7
	*ctl-1*	Catalase^b^	
	*ctl-3*	Catalase^b^	
Xenobiotic degradation and detoxification	*cdr-3*	Glutathione *S*-transferase	493
	*djr-1.2*	Glyoxalase	185
	*alh-2*	Aldehyde dehydrogenase	77
	*glod-4*	Glyoxalase	24 (2.8)
	*cdr-2*	Glutathione *S*-transferase	6.7
Heat shock stress response	*F08H9.4*	HSP-16-like protein	704
	*F08H9.3*	HSP-16-like protein	40
	*hsp-70*	Heat shock protein	6
	*hsp-12.6*	αB-crystallin	3.2 (2.9)
Intrinsically disordered proteins	*lea-1*	Late embryogenesis abundant-like protein	3.8 (7.1)
	*dur-1*	Dauer upregulated protein	2.1 (1.7)
Fatty acid desaturation	*fat-6*	∆9 Fatty acid desaturase	14
	*cyp-33C9*	Cytochrome P450-family member	5
	*fat-4*	∆5 Fatty acid desaturase	5.4
	*fat-5*	∆9 Fatty acid desaturase	2.6
	*fat-1*	ω3 Fatty acid desaturase	N/S^c^
	*fat-3*	∆6 Fatty acid desaturase	N/S
	*fat-7*	∆9 Fatty acid desaturase	N/S
Polyamine biosynthesis	*odc-1*	Ornithine decarboxylase	N/S
	*spds-1*	Spermidine synthase	N/S
Hygrosensation	*osm-11*	Osmosensitivity-related protein	2.1 (2.1)
	*daf-6*	Patched-related protein	N/S
	*osm-9*	TRPV channel protein	N/S
	*ocr-2*	TRPV channel protein	-1.3
	*ocr-3*	TRPV channel protein	-1.6
	*ocr-1*	TRPV channel protein	-2.5
Novel proteins	*try-5*	Trypsin-like protease	27
	*C04G2.2*	Serine/threonine kinase	21
	*cex-1*	Calexcitin	10 (1209)
	*ugt-1*	UDP-glucuronosyltransferase	6.5
	*cex-2*	Calexcitin	N/S

Fold changes are presented according to microarray results. When applicable, figures in parentheses indicate the fold changes according to geLC-MS/MS analyses. Catalases were shown to increase only by 2D-DIGE analysis, therefore their fold changes are not calculated. N/S: Not significant.

Survival data were analyzed using beta regression [34]. The estimated survival rates and their approximate standard errors are presented in Table S2. We categorized the desiccation sensitivity phenotype of mutants according to their survival rates into 4 groups. Any strain whose survival rate was not significantly different from the control (p ≥ 0.05) was considered as desiccation tolerant. The others were categorized as desiccation sensitive (> 50% survival), very sensitive (25 - 50% survival) and extremely sensitive (< 25% survival).

### A. Small HSPs Are Essential for Desiccation Tolerance

According to microarray and proteomics surveys, several HSPs were highly upregulated upon desiccation stress (Datasets S1, S3). The gene with the overall highest differential expression (704-fold), *F08H9.4*, encodes an HSP-16-like protein. 

Various types of abiotic stress may cause protein aggregation, which is eliminated by the cell via molecular chaperones known as HSPs, and by proteases when necessary. HSP90, HSP70, and chaperonins act as molecular chaperones via ATP hydrolysis [35], whereas small HSPs and α-crystallins bind proteins in non-native conformations in an ATP-independent manner [36]. Certain HSPs were first implicated in plant desiccation tolerance [37], then also in the anhydrobiotic African chironomid [18], a tardigrade [38], and a nematode [39].

**Figure 3:**
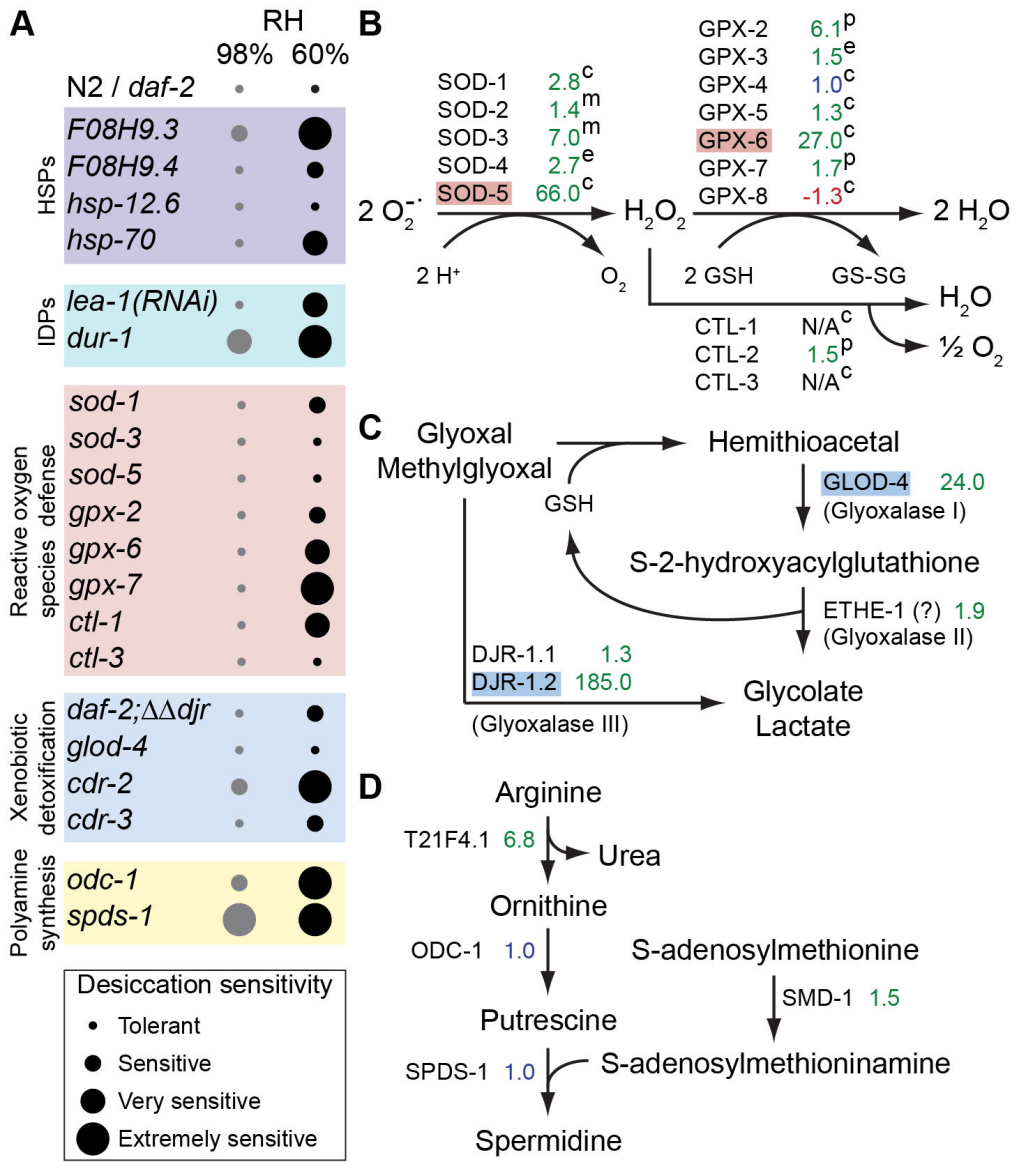
Major pathways involved in desiccation tolerance. (A) Desiccation sensitivities of various mutants from (B-D) candidate pathways are presented in 4 categories. Genes from high FCC are highlighted according to Figure 1. The subcellular localizations of ROS defense enzymes are shown as c, cytosol; e, extracellular; m, mitochondrion; and p, peroxisome.

*F08H9.3*, *F08H9.4* and *hsp-70* mutants were desiccation sensitive (Figure 3A). A particularly strong phenotype was observed in the HSP-16-like protein mutant *F08H9.3*, which was already affected during preconditioning. Remarkably, despite the redundancy of genes in this class, disruption of a single HSP gene still induced a strong phenotype. We conclude that HSPs, especially small HSPs, are essential for desiccation tolerance in *C. elegans*.

### B. Desiccation Response via LEA-like Protein Expression

LEA-1, a *C. elegans* homolog of plant LEA proteins, was the major protein whose abundance increased upon preconditioning in 2D-DIGE gels (Figure 2, S2C). Microarray and geLC-MS/MS approaches also detected LEA-1 as a gene/protein that was highly expressed during desiccation (Datasets S1, S3). LEA proteins are associated with desiccation tolerance in plant seeds, as well as the vegetative tissues of anhydrobiotic plants. Recently, LEA homologs were discovered in many animal species [17]. Classified as IDPs, LEA proteins obtain their functionally active conformations upon desiccation, unlike other proteins [40]. They can reduce protein aggregation by lessening the desiccation-induced interactions between proteins and polypeptides, therefore acting as molecular shields [41,42]. 

Because the *lea-1* mutant is not available, we performed the desiccation assay using RNAi. *lea-1-*knockdown worms were very sensitive to desiccation at 60% RH but not at 98% RH, unlike it was previously reported [43] (Figure 3A). Given that RNAi seldom fully eliminates the target mRNA, it can be concluded that LEA-1 protein is essential for desiccation tolerance.

In addition to *lea-1*, *C. elegans* possesses another gene, *dur-1*, which encodes a homolog of rotifer (*Adineta ricciae* and *Adineta vaga*) LEA proteins (Figure S3). DUR-1 is also similar to *C. elegans* LEA isoforms, as well as LEA isoforms from two other nematodes (*Caenorhabditis remanei* and *Caenorhabditis briggsae*) and some plants (*Medicage truncatula* and *Arabidopsis lyrata lyrata*). *dur-1* mutants were extremely sensitive to desiccation at 60% RH (Figure 3A). Furthermore, these mutants were already very sensitive to mild desiccation, which suggests that DUR-1 is required already during preconditioning. Altogether, these results clearly indicate that IDPs are essential for the anhydrobiotic response.

### C. Desiccation Tolerance Requires Cytoplasmic ROS Defense

Another prominent pathway revealed by the microarray and proteome data was ROS defense. Biochemical reactions that use electron transfer to molecular oxygen (such as respiration and photosynthesis) naturally cause the formation of ROS. These are highly reactive molecules, which can damage proteins, lipids, and DNA inside cells. Therefore, cells have enzymatic and non-enzymatic mechanisms to prevent the formation of ROS or to repair the damage caused by them. A major ROS is the superoxide radical (O_2_^–^), which is produced by the transfer of a single electron to molecular oxygen (Figure 3B). Superoxide dismutases (SODs) can convert O_2_^–^ into hydrogen peroxide (H_2_O_2_) and oxygen (O_2_). However, H_2_O_2_ is itself an ROS and should be inactivated. Catalases (CTLs) can directly convert H_2_O_2_ into H_2_O and O_2_. Alternatively, glutathione (GSH) peroxidases (GPXs) can convert H_2_O_2_ into H_2_O via reducing GSH [16] (Figure 3B). In addition to SODs, CTLs, and GPXs, worms have peroxiredoxins, glutaredoxins, thioredoxins, and thioredoxin reductases that participate in ROS detoxification. Our microarray survey showed that all SODs, five GPXs, and CTL-2, as well as some peroxiredoxins, glutaredoxins, thioredoxins, and thioredoxin reductases were upregulated upon desiccation stress (Figure 3B, Dataset S1). Two of these genes (*sod-5* and *gpx-6*) were in the high FC class. Furthermore, 2D-DIGE analysis showed that CTL-1 and CTL-3 proteins were strongly induced during the dauer transition and were upregulated by preconditioning (Figures S2A, S2D).

The desiccation assay showed that worms with mutated components of the ROS defense pathway, namely a cytosolic SOD (SOD-1) [44], two peroxisomal GPXs (GPX-2 and GPX-7) [45], a cytosolic GPX (GPX-6), and a cytosolic CTL (CTL-1), were sensitive to desiccation at 60% RH (Figure 3A). However, all of the mutants were desiccation tolerant at mild humidity (98% RH). Similar to the small HSP pathway, ROS defense genes are redundant. Despite this, *gpx-7* mutant was extremely desiccation sensitive (Figure 3A). These results indicate that cytosolic and peroxisomal ROS defense have an essential role in desiccation tolerance.

### D. Desiccation Induces Detoxification Mechanisms

Among the upregulated genes of the high FCC in the microarray survey were two genes encoding glyoxalases: *djr-1.2* and *glod-4* (Figure 1B). The main function of glyoxalases is to detoxify α-oxoaldehydes such as glyoxal (GO) and methylglyoxal (MGO), which are byproducts of glycolysis [46] (Figure 3C). These molecules react with the amino groups of proteins and form advanced glycation end-products, and thereby cause protein damage. In mammals, GO and MGO are detoxified by the glyoxalase I/II system in a GSH-dependent manner [47]. In *C. elegans*, GLOD-4 protein is a glyoxalase I and decreases mitochondrial ROS production [48]. A novel type of glyoxalase homolog, glyoxalase III, was recently discovered in humans, mice, and worms [49]. This enzyme, DJ-1 (also named PARK7 because it has been associated with early-onset Parkinson’s disease [50]) is GSH-independent and can directly detoxify GO and MGO. *C. elegans* has two genes, *djr-1.1* and *djr-1.2*, that encode DJ-1 isoforms [49]. Deletion of both of these genes on *daf-2* background (*daf-2;∆∆djr*) rendered the worms sensitive to desiccation (Figure 3A). However, single mutants of *djr-1.1*, *djr-1.2* or *glod-4* did not exhibit desiccation sensitivity. This is most likely due to the redundant functions of glyoxalase systems. Further investigation of *djr-1.1;djr-1.2;glod-4* mutants will help us understand better the role of glyoxalase activity on desiccation tolerance.

Other xenobiotic degradation mechanisms might also be required for anhydrobiosis. Cadmium (Cd) toxicity is a xenobiotic stress for *C. elegans*. A novel Cd-responsive gene, *cdr-1*, and its six paralogs (*cdr-2*–*7*) have previously been identified [51,52]. Cd treatment induces transcription of *cdr-1* by more than 50-fold. However, the other *cdr* genes are not induced as dramatically as *cdr-1* [52]. All these proteins are predicted to be transmembrane [52] and to contain putative GST domains (Table S3). Our microarray survey revealed that the levels of all *cdr* transcripts, except *cdr-1* and *cdr-5*, significantly increased upon desiccation stress. Mutation of *cdr-2* results in extreme desiccation sensitivity at 60% RH, whereas mutation of *cdr-3* has a milder effect (Figure 3A). These results suggest that the detoxification function of *cdr* genes may have a role in desiccation tolerance.

### E. Polyamine Biosynthesis as an Anhydrobiotic Strategy in Animals

According to the geLC-MS/MS analysis, the level of the T21F4.1 protein was very strongly elevated during preconditioning (Dataset S3). Furthermore, in the microarray survey, this gene was found in the medium FCC. T21F4.1 is an ortholog of human arginase 1, which is mainly involved in the urea cycle and is partially involved in the biosynthesis of polyamines. Polyamines, namely putrescine, spermidine, and spermine, are polycations that are associated with diverse functions in the cell. As in other organisms, the first step of this pathway in *C. elegans* is the conversion of arginine into the amino acid ornithine by the mitochondrial enzyme arginase [53] (Figure 3D). Subsequently, ODC-1 produces putrescine by decarboxylation of ornithine [54], which is then converted into spermidine by SPDS-1 [55] (Figure 3D).

We tested available strains bearing mutations in genes of this pathway. Although levels of SPDS-1 and ODC-1 mRNA did not change upon desiccation according to the microarray survey, the effect of *odc-1* and *spds-1* mutations on the desiccation tolerance of dauer larvae was very strong (Figure 3A). In fact, *spds-1* mutants were extremely sensitive to desiccation already at 98% RH. These results suggest that polyamines, especially spermidine, are required for desiccation tolerance.

Recently, polyamine back-conversion (i.e., spermine to putrescine) via polyamine oxidases was implicated in drought stress in plants [56]. Our microarray survey showed a 2.8-fold upregulation of the amine oxidase gene *amx-2* (Dataset S1), which has homology to polyamine oxidases from various organisms. Thus, along with spermidine biosynthesis, putrescine back-conversion might be an essential anhydrobiotic strategy used by *C. elegans*. However, the molecular details of this strategy remain to be investigated.

### F. Role of Polyunsaturated Fatty Acids in Desiccation Tolerance

**Figure 4:**
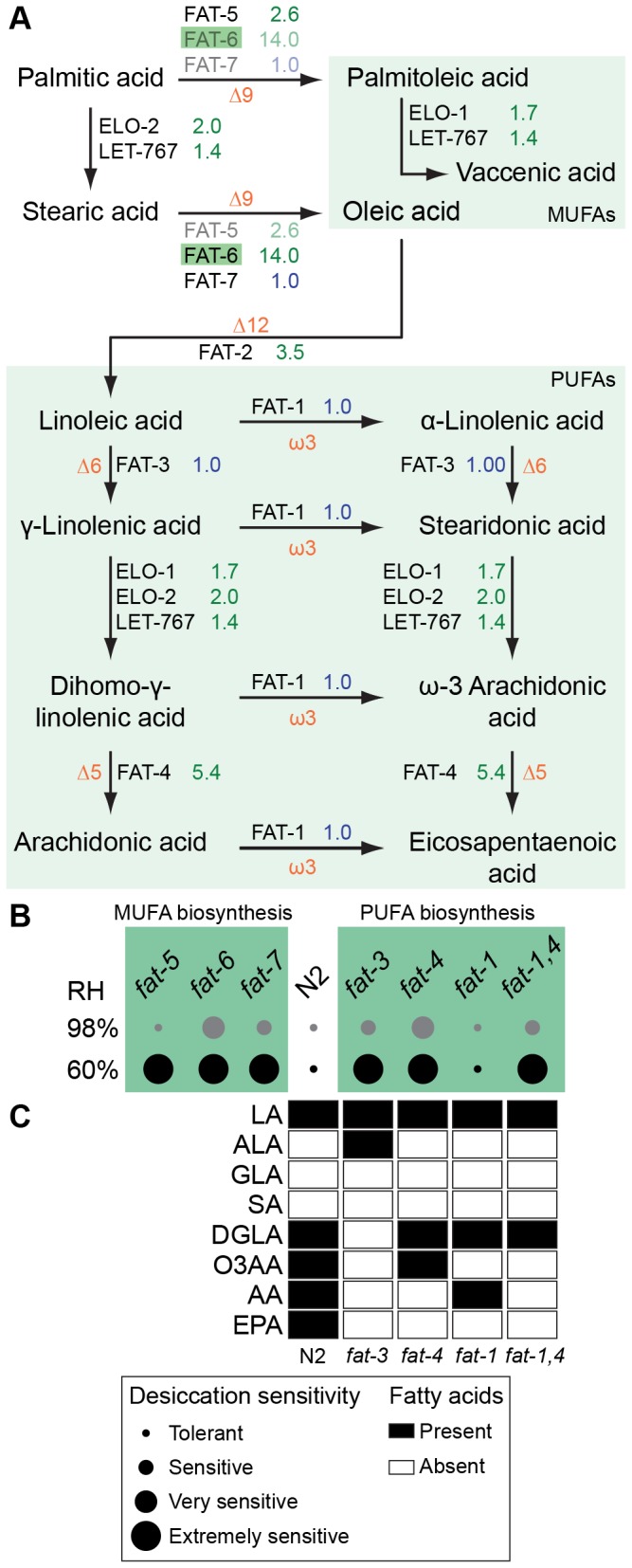
Arachidonic acid is essential for desiccation tolerance. (A) The major components in the biosynthesis of MUFAs and PUFAs in *C*. *elegans* (highlighted according to Figure 1). Type of desaturation is shown in orange for every reaction. Modified from [57,59]. (B) Desiccation sensitivities of FAT mutants in 4 categories. (C) PUFA profiles of preconditioned dauer larvae of wild-type worms and fat mutants. Filled and empty boxes indicate the presence and absence of the PUFA species, respectively. LA, Linoleic acid; ALA, α-linolenic acid; GLA, γ-linolenic acid; SA, stearidonic acid; DGLA, dihomo-γ-linolenic acid; O3AA, ω-3 arachidonic acid; AA, ω-6 arachidonic acid; EPA, eicosapentaenoic acid.

Preconditioning induces several lipid-binding proteins and lipid-modifying enzymes (cf. subsection G below). Among these is the class of FATs, which produce monounsaturated FAs (MUFAs) from dietary or *de novo* synthesized palmitic acid, and subsequently polyunsaturated fatty acids (PUFAs) from MUFAs (Figure 4A) [57]. The *C. elegans* genome has seven genes (*fat-1–7*) that encode five types of FATs. Among these, FAT-5, FAT-6, and FAT-7 can redundantly catalyze ∆9 desaturation [58]. This gives rise to MUFAs, among which oleic acid (C18:1n-9) is further desaturated into linoleic acid (C18:2n-6) by FAT-2. This is the first PUFA and is a precursor to all other PUFAs in subsequent desaturation and elongation reactions [59]. The end product of this pathway is eicosapentaenoic acid (EPA, C20:5n-3), the major PUFA in *C. elegans* [57,60] (Figure 4A).

Preconditioning induced ∆9 (FAT-5 and FAT-6), ∆12 (FAT-2), and ∆5 (FAT-4) FATs, as well as FA elongases (ELO-1, ELO-2, and ELO-9) (Figure 4A, Dataset S1). We first tested the desiccation tolerances of the ∆9 desaturase mutants *fat-5*, *fat-6*, and *fat-7*. All these mutants were extremely sensitive to desiccation at 60% RH (Figure 4B). In *fat-6* and *fat-7* mutants, desiccation sensitivity appeared already at 98% RH. Remarkably, single mutants do not have any detectable phenotypes under normal conditions and are superficially wild-type [57]. By contrast, all three ∆9 desaturases are essential during desiccation. An explanation for this could be that FATs are differentially expressed in various cells. Thus, the viability of the cell is not affected in the absence of unsaturated FAs under normal conditions; however, desaturation seems to be essential for desiccation tolerance.

∆9 FA desaturation is the first step in the production of PUFAs. Are PUFAs required for the desiccation tolerance, similar to MUFAs? To address this question, we tested *fat-3*, *fat-4*, *fat-1*, and *fat-4*;*fat-1* (designated as *fat-1,4*) mutants in the desiccation assay. Except for *fat-1*, all mutants were extremely desiccation sensitive at 60% RH. (Figure 4B). The same mutants were affected by mild desiccation as well. Thus, we conclude that not only MUFAs but also PUFAs are required for desiccation tolerance.

The schematic in Figure 4C shows the FA contents after preconditioning (as detected by us in the dauer larva, similar to non-preconditioned reproductive stage worms [61]). The correlation between the FA contents and desiccation resistance phenotypes suggests that worms require C20 PUFAs (because the *fat*-3 mutant was extremely sensitive to desiccation) and, in particular, ω-6 arachidonic acid (AA) (because the *fat-4* and *fat-*1,4 mutants were extremely sensitive, whereas the *fat-1* mutant was desiccation tolerant). This suggests that AA is an essential PUFA for desiccation tolerance. 

In addition to MUFAs and PUFAs, *C. elegans* can also produce hydroxy- and epoxy-derivatives of AA and EPA [62], which most probably act in signal transduction. One of the genes involved in this process, *cyp-33C9*, was significantly upregulated in response to desiccation in our microarray survey. When tested in the desiccation assay, the *cyp-33C9* mutant displayed desiccation sensitivity, although this phenotype was much less severe than that of the *fat* mutants (Table S2). This suggests that not only AA (and possibly EPA) but also their hydroxy- and epoxy-derivatives may be required for desiccation tolerance. 

### G. Other Novel Strategies for Desiccation Tolerance

**Figure 5:**
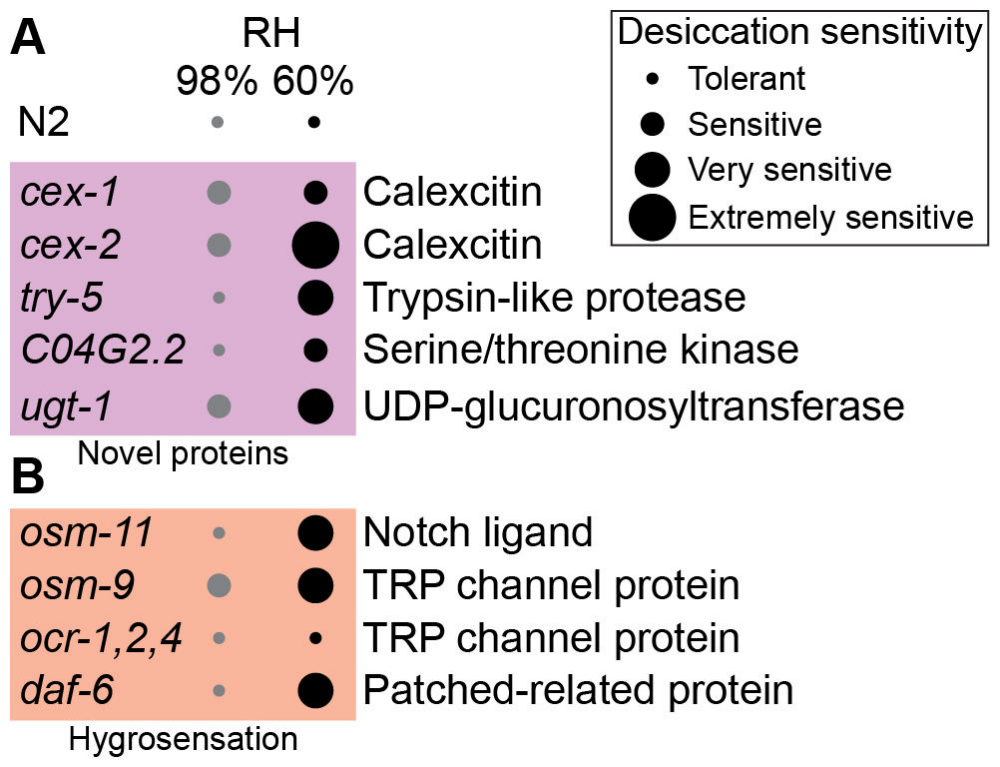
Novel DTR proteins and putative elements of the hygrosensation pathway involved in desiccation tolerance.

In addition to those mentioned above, we chose mutants of other annotated genes that were induced by preconditioning, according to our transcriptome and proteome data. The desiccation tolerances of these mutants were tested (Table 1). Among them, *cex-1*, *cex-2*, try-*5*, *ugt-1*, and *C04G2.2* mutants showed sensitivity to desiccation at 60% RH (Figure 5A). Furthermore, *cex-1, cex-2* and *ugt-1* mutants were desiccation sensitive already at 98% RH.

*cex-1* and *cex-2* are *C. elegans* homologs of the Ca-binding protein calexcitin, which putatively regulates membrane excitability in neurons [63] and muscle cells [64]. try-*5* has recently been identified as an extracellular sperm-activating protease [65]. *ugt-1* is a UDP-glucosyltransferase that is genetically activated by *daf-16* [66]. Its closest human ortholog, UGT1A6, is involved in phenol detoxification [67]. C04G2.2 is a nematode-specific tau tubulin kinase-like protein [68]. However, knockdown of this gene does not significantly affect the life span of the animal [66].

In addition to novel DTR genes with known function, we found many uncharacterized genes in the high FCC. To deduce the functions of the proteins encoded by these genes in desiccation tolerance, we analyzed their sequences using various bioinformatics tools (Table S4).

Nematodes often display many uncharacterized genes that lack clear orthologs in higher organisms; therefore, we focused on the conserved domains of the proteins of interest. In four proteins from the high FCC (F11F1.4, F11F1.5, F11F1.8, and F13E9.14), a domain of unknown function (DUF148) was found, which seems to be nematode-specific (Figure S4A). Hierarchical clustering of expression levels of high FCC proteins showed that three of these paralogs (F11F1.4, F11F1.5, and F11F1.8) have a very similar differential expression pattern (Figure 1B). Homology detection and structure prediction by HMM-HMM comparison (HHPRED) of DUF148 revealed similarity to a proteobacterial domain (Lipase_chap, PF03280) that is present in lipase chaperones [69] (Figure S4B). These chaperones are required for functionally active lipases [70]. DUF148 lacks part of the N-terminal sequence of the Lipase_chap domain (Figure S4B), which suggests that the molecular functions of DUF148-containing proteins might be different from those of their proteobacterial orthologs. Overall, this finding suggests that desiccation tolerance requires a wide array of chaperones, including HSPs, IDPs, and lipase chaperones. Notably, all four DUF148 proteins contain an N-terminal signal peptide, and thus could be secreted. One of them, F13E9.14, has in addition several YGG-repeats, which are also found in the P granule proteins PGL-1 and GLH-1 in *C. elegans* embryos [71].

As a next step to understand the possible function of the uncharacterized high FCC proteins, we searched for their potential interacting partners by using the Search Tool for the Retrieval of Interacting Genes/Proteins (STRING) [72]. Figure S5 shows all identified molecular interactors of four uncharacterized high FCC proteins. Among these, R05D7.2 seems to be associated with the nucleolar RNA processing machinery (Figure S5A). F53A9.2 may be involved in carbohydrate metabolism (Figure S5B) and C40H1.3 is associated with a SUMO ligase (Figure S5C). Finally, C54F6.5 and F41C3.1 are connected to the tumor suppressor and E3 ubiquitin ligase VHL-1, as well as a cytochrome P450 (Figure S5D).

As mentioned before, desiccation stress might induce post-translational modifications of proteins. Via mass spectrometric analysis of gel-isolated spots, we found that myosin light chain proteins MLC-1 and MLC-2 were dephosphorylated during preconditioning (Figures 2, S2E). This modification was reversed by rehydration (Figures S2B, S2E). Furthermore, the actin isoform ACT-4 was possibly phosphorylated during preconditioning (Figures 2, S2E) and rehydration (Figure S2E). These data imply that certain post-translational modifications of cytoskeletal proteins may be required in the anhydrobiotic state. Therefore, cytoskeletal reorganization could be another molecular strategy of anhydrobiosis in *C. elegans*.

### The Desiccative Environment Might Be Sensed by Head Neurons

All our data indicate that mild desiccation stress (preconditioning) induces differential expression of many genes at the mRNA and protein levels. In this way, the organism is prepared for an anticipated harsher desiccation condition. Thus, dauer larvae should be able to detect changes in humidity and have mechanisms to convert the desiccation signals into changes at the protein expression level.

Sensing of the osmolarity of the environment has been thoroughly studied in *C. elegans* [73-76]. We speculated that sensing of humidity could involve similar genes and molecular mechanisms. Indeed, it was gratifying to find that *osm-11* and its paralog *dos-1* were significantly induced upon desiccation stress (Datasets S1 and S3). *osm-11* is one of the *osm* genes (OSMotic avoidance abnormal) that are required for osmotic avoidance [76]. OSM-11 is a Notch ligand in seam cells and acts on Notch receptors in head neurons [77]. Together, OSM-11 and DOS-1 are required for avoidance of 1-octanol [77]. *osm-11* mutants were very sensitive to desiccation at 60% RH (Figure 5B). This suggests that hygrosensation might be associated with certain head neurons. The role of one such neuron, ASH, in osmosensation is well established [74]. ASH neurons express TRP channels formed by OSM-9 and OCR-2, which are associated with various sensory functions [78,79]. We performed the desiccation assay using *osm-9* and *ocr-4;ocr-2;ocr-1* (designated as *ocr-1,2,4*) mutant dauers. *osm-9* mutants were very sensitive to desiccation although *ocr-1,2,4* mutants were desiccation tolerant (Figure 5B). Thus, hygrosensation and osmosensation might involve similar molecular mechanisms.

To corroborate our results on the involvement of head neurons in hygrosensation, we investigated whether this process requires intact amphids. The amphids on the head of the worm are openings through which sensing neurons are exposed to the environment. ASH neurons extend their cilia to the amphid. Deletion of the patched-related gene *daf-6* affects amphid formation such that amphid neurons cannot reach the exterior. This results in insensitivity to chemical signals, including the dauer pheromone (thereby leading to a dauer formation deficiency phenotype) [80,81]. For the desiccation experiments, we exploited the fact that *daf-6* mutants can form proper dauers if cholesterol is substituted by lophenol for two generations [82]. *daf-6* dauers were very sensitive to desiccation at 60% RH (Figure 5B). This result, together with the aforementioned results, underlines the importance of head neurons in hygrosensation. 

## Discussion

In this study, we started to investigate the molecular strategies of *C. elegans* dauer larva to survive severe desiccation. Perhaps the most striking result of our survey is that the desiccation response is very focused and involves a relatively small number of genes and functional pathways. Only about 2.5% of the surveyed genes show medium (444 genes) or high (63 genes) levels of differential expression. In addition, these genes are often paralogs of the same protein family, rendering the number of functional pathways even smaller. The diversity of proteins synthesized upon preconditioning, as revealed by geLC-MS/MS or 2D-DIGE, is even lower. Furthermore, the majority of the mutants that exhibited a phenotype in the desiccation assay were superficially wild type under normal conditions. Thus, the desiccation response induces a set of specific proteins that are probably not essential for housekeeping functions.

Another feature is that during entry into anhydrobiosis, worms invest in preserving existing structures rather than in building new ones. These features of the response could be explained by the narrow time window during which the organism has to react to humidity changes. Transcription and translation can only occur in aqueous milieu; therefore, organisms should produce only essential proteins before water loss makes these processes impossible. Moreover, to save time, dauer larvae might produce DTR proteins using existing mRNAs. This could explain the massive production of LEA-1, which dominates all other proteins, as revealed by 2D-DIGE, despite its mRNA level only increasing moderately.

**Figure 6:**
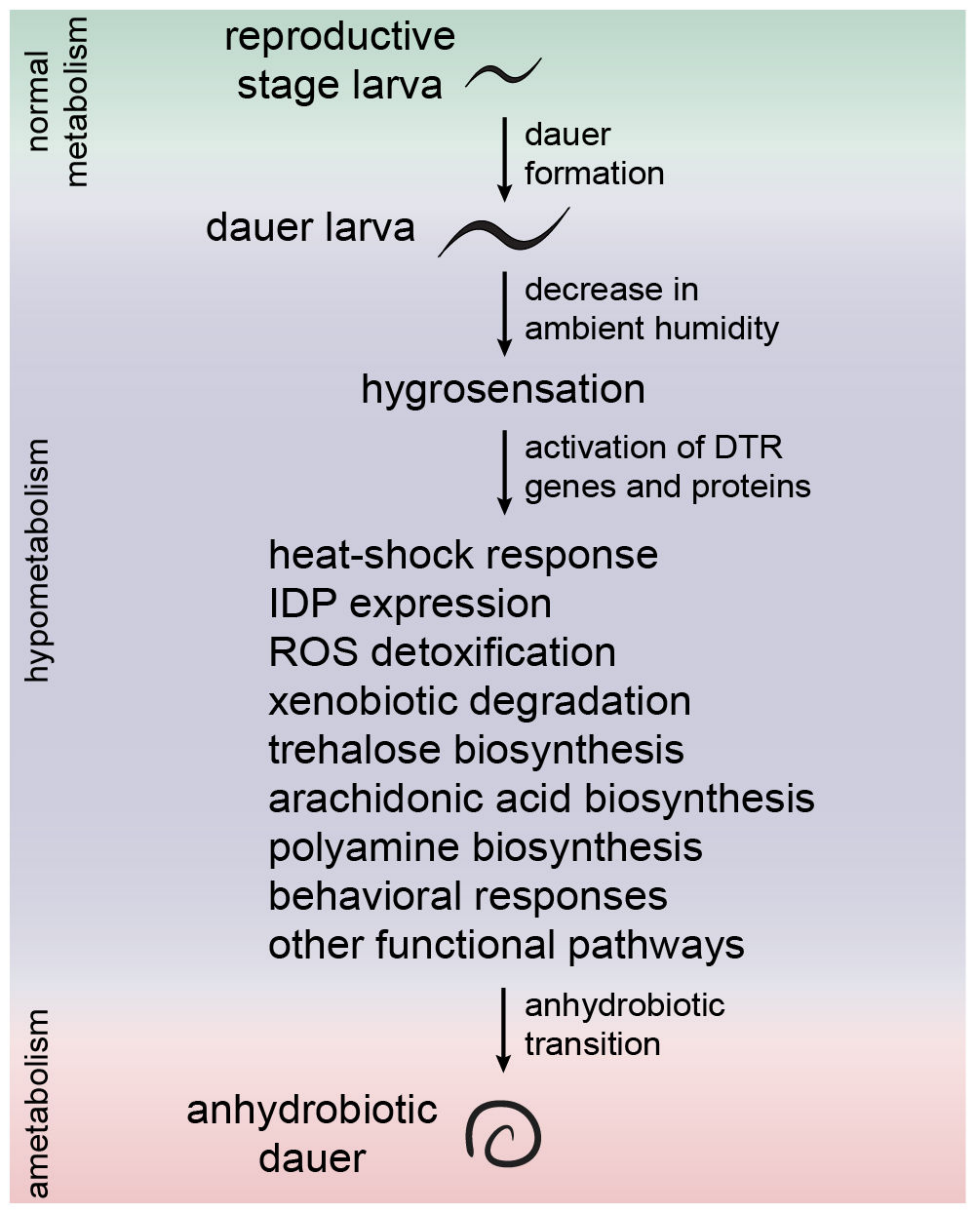
Suggested model of the main strategies of desiccation tolerance in *C*. ***elegans***. The hypometabolic dauer larva senses a decrease in ambient humidity, perhaps via head neurons, and initiates a desiccation response at different levels. As a result of this, ROS and xenobiotics are eliminated, proteins and membranes are stabilized, and other essential functions are fulfilled. The ametabolic transition (anhydrobiosis) can only succeed under these conditions.

What are the key aspects of desiccation tolerance? An overview of the suggested strategies in the dauer larva is depicted in Figure 6. The first decisive phase of the desiccation response is hygrosensation. Neither the physical basis of the sensation nor the putative receptors are known. Our data suggest that humidity changes may be sensed centrally via head neurons. Similar to osmosensation, the response requires intact amphids. Several proteins involved in osmosensation, such as TRP channels, might also be involved in hygrosensation, which makes the *C. elegans* response similar to that of Drosophila [25]. The signal detected by head neurons can be translated into behavioral and biochemical responses via activation of DTR genes and proteins, possibly by an unidentified transcription factor. The combined action of these genes and proteins should result in increased protein and membrane stability, ROS and xenobiotic detoxification, production of osmolytes, and induction of some other functional pathways. After these requirements are fulfilled, the organism can successfully enter the ametabolic state, anhydrobiosis.

One of the major problems for a dehydrating organism is the misfolding and aggregation of proteins. Organisms have evolved mechanisms against desiccation-induced protein aggregation, such as expression of HSPs [18,38] or IDPs [11,17,22,41,42,83,84]. Our data demonstrate that *C. elegans* uses both of these mechanisms. The reduced desiccation tolerances of *F08H9.3, F08H9.4*, *hsp-70*, and *dur-1* mutants and worms treated with *lea-1* RNAi suggest that HSP- and IDP-mediated proteostasis is essential for anhydrobiosis in *C. elegans*.

Desiccation might also be connected to several toxic processes, including the production of ROS as byproducts of oxidative phosphorylation (and photosynthesis in plants). Although the source is unknown, evidence suggests that ROS levels increase during desiccation in yeast [85]. Similarly, a mechanism that is probably conserved between plants and animals is increased ROS defense activity in response to desiccation stress [7,86-89]. Our data clearly show that ROS defense is activated at the transcriptional and translational levels in response to desiccation stress. Mutations of cytosolic and peroxisomal ROS defense enzymes strongly decrease desiccation tolerance. Although the molecular mechanisms of ROS formation and regulation during desiccation stress in *C. elegans* remain unknown, ROS defense might have an important role in anhydrobiosis.

ROS may not be the only cause of toxicity in the desiccated state. Our data suggest that the glyoxalase genes *djr-1.2* and *glod-4* are highly upregulated during preconditioning. Mutation of *djr-1.2* and its paralog *djr-1.1* at the same time decreased desiccation tolerance. Because DJR-1.2 is a cytoplasmic protein localized mainly to neurons [49] and induced in the dauer stage [90], we suggest that neuronal 2-oxoaldehyde detoxification may be an essential strategy used by the *C. elegans* dauer to survive desiccation. Remarkably, *djr-1.1* and *djr-1.2* are homologs of the Parkinson’s disease-associated human gene DJ-1, also known as PARK7 [49,50]. This homology is encouraging and indicates that DJ-1 might have a protective role, which might assist the search for a treatment for early-onset Parkinson’s disease. 

Another detoxification mechanism involves GST proteins [91], which can neutralize xenobiotics. Many genes encoding GST-domain proteins were induced by desiccation, among which was a class known as Cd-responsive genes (Table S3). The first member of this class, *cdr-1*, is strongly and specifically induced by Cd [51], although the other members of this class are not affected to the same extent [52]. In contrast to Cd stress, desiccation does not induce *cdr-1* expression but strongly induces *cdr-2, -3, -4*, and -6. This suggests that CDRs could be a novel class of desiccation tolerance proteins in *C. elegans*.

In addition to proteins, desiccation clearly challenges membranes [1,19,20]. FAs are essential components of the membrane structure, meaning their properties might determine the stability of phospholipid bilayers under desiccative conditions. One such property is the desaturation of FAs. Our data indicate that the unsaturated FA biosynthesis pathway is upregulated overall in response to desiccation stress. Upregulation of *fat-2* has also been detected in another nematode under desiccation and osmotic stresses [92]. Our functional analyses of *fat* mutants based on their desiccation sensitivities (Figure 4B) and free PUFA profiles (Figure 4C) strongly indicate that arachidonic acid is a novel, major PUFA associated with anhydrobiosis.

To our surprise, proteome analysis of the worm showed an almost 200-fold increase in the level of arginase (T21F4.1), which converts arginine into ornithine. We speculated that the role of arginase in the desiccation response could be associated with polyamines because ornithine is the source of putrescine and spermidine. Although the enzymes that synthesize polyamines, namely ornithine decarboxylase ODC-1 and spermidine synthase SPDS-1, were not upregulated, mutants displayed dramatic sensitivity to desiccation. Polyamines accumulate upon various biotic (pathogens) and abiotic (draught, salinity, high temperature, UV, xenobiotics, etc.) stresses in plants [56,93]; however, their functions during desiccation remain elusive. Here, we show that polyamines are essential for animal anhydrobiosis. *C. elegans* could prove to be a very useful model organism in which to study the molecular mechanisms involving polyamines in stress conditions.

As exemplified by polyamine biosynthesis, some essential anhydrobiotic pathways may not be revealed by transcriptome and proteome analysis. Therefore, in addition to the pathway-oriented approach, we also began a systematic search for anhydrobiosis-related genes based on our microarray and proteomics results. This approach revealed some essential anhydrobiosis-related genes from various classes (Figure 5A). Moreover, we focused on the uncharacterized proteins found in the high FCC to identify putative novel anhydrobiotic strategies. Remarkably, four proteins with a domain of unknown function (DUF148), which is similar to proteobacterial lipase chaperone domains (Figure S4B), were found in this category. This suggests that not only HSPs and IDPs but also other types of chaperones may be implicated in anhydrobiosis.

Anhydrobiosis is a state of ametabolism. To the best of our knowledge, the dauer larva is the only developmental stage of *C. elegans* that is capable of undergoing anhydrobiosis [19]. It is widely accepted that the metabolic traits of dauer and reproductive larvae greatly differ [94-96]; the former displays predominantly low level anaerobic metabolism, i.e., hypometabolism. Thus, dauer formation itself could be considered to be preparation for anhydrobiosis, and the transition from full metabolism to ametabolism can occur only via the hypometabolic stage. Losing water in the state of full metabolism may induce uncontrolled biochemical activity due to increased concentrations of enzymes and substrates. Therefore, it may be more favorable for the organism to turn off all metabolic pathways, except for basal ones, so that metabolism can be completely halted. Such a gradual decrease in metabolism has been suggested in anhydrobiotic *Artemia* eggs [97], so that the dehydrating organism first enters a restricted metabolic state at a critical hydration level. Further dehydration beyond another critical level induces total metabolic shutdown. Additionally, only stationary phase yeast cells are desiccation-tolerant in a cell-autonomous manner [6,98,99], which implies that the yeast ametabolic transition is not possible in a fully active metabolic state. We suggest that the counterpart of restricted metabolism in *C. elegans* may be the dauer state, which could explain why only dauer larvae can undergo anhydrobiosis.

In summary, we present essential, core strategies that *C. elegans* might exploit to resist desiccation. Certainly, other functional pathways will be identified in the future. However, each of the functional pathways described here requires detailed investigation. Our study demonstrates that the worm is an excellent model organism in which to study anhydrobiosis at the organismal, cellular, and molecular levels by using established genetic, biochemical, and biophysical tools. Thorough understanding of the anhydrobiotic ability of the worm will enable engineering of anhydrobiotic cells, tissues, and perhaps even organisms.

### Materials and MethodsWorm Cultures and Strains

*C. elegans* wild-type (N2), *C54E4.2*(*gk1083*);*alh-2*(*gk3053*), *cdr-2*(*ok1996*), *cdr-3*(*ok864*), *cex-1*(*ok3163*), *cex-2*(*ok2767*), *ctl-1*(*ok1242*), *ctl-3*(*ok2042*), *daf-2*(*e1370*), *daf-6*(*e1377*), *dur-1*(*ok1010*), *F08H9.4&srz-97*(*ok1976*), *fat-1*(*ok2323*), *fat-3*(*wa22*), *fat-4*(*ok958*), *fat-4*(*wa14*);*fat-1*(*wa9*), *fat-5*(*tm420*), *fat-6*(*tm331*), *fat-6*(*tm331*);*fat-5*(*tm420*), *fat-6*(*tm331*);*fat-7*(*wa36*), *fat-7*(*wa36*), *fat-7*(*wa36*);*fat-5*(*tm420*), *hsp-12.6*(*ok1077*), *ocr-4*(*vs137*);*ocr-2(ak47*);*ocr-1*(*ok132*), *odc-1*(*pc13::Tc1*), *osm-9*(*ok1677*), *osm-11*(*rt142*), *sod-1*(*tm776*), *sod-3*(*gk235*), *sod-5*(*tm1146*), *spds-1*(*ok3421*), and *ugt-1*(*ok2718*) strains were obtained from *Caenorhabditis* Genetics Center, USA, which is funded by the NIH Office of Research Infrastructure Programs (P40 OD010440). *C04G2.2*(*tm3841*), *cyp-33C9*(*tm3809*), *djr-1.1*(*tm918*), *djr-1.2*(*tm951*), *F08H9.3*(*tm5012*), *hsp-70*(*tm2318*), *glod-4*(*tm1266*), *gpx-2*(*tm2895*), *gpx-6*(*tm2535*)*, gpx-7*(*tm1990*), and try-*5*(*tm3813*) strains were obtained from the National Bioresource Project, Japan. *C04G2.2, cex-1, cex-2, daf-2, daf-6, djr-1.1, djr-1.2, dur-1, F08H9.4, fat-3, fat-4, fat-5, fat-6, fat-7, gpx-2, gpx-7, hsp-70, odc-1, osm-9, osm-11, sod-1, sod-5,* try-*5* and *ugt-1* mutants were outcrossed with the wild type before or during functional analyses to eliminate the possibility that a background mutation causes the desiccation sensitivity phenotype.

Worms were maintained on NGM agar plates seeded with *Escherichia coli* NA22 at 15 °C [100]. Large quantities of dauers for RNA and protein extraction were obtained by growing *daf-2* eggs at 25 °C in liquid culture [101]. *daf-2* L3 larvae were also produced the same way, only this time by growing at 15 °C. Small quantities of dauers for desiccation assays were grown on sterol-depleted lophenol-substituted agarose plates in two generations [82] for all strains except *daf-2* and *daf-2;∆∆djr*. Gene silencing via RNAi was performed by growing *daf-2* eggs into dauers at 25 °C on *E. coli* HT115(DE3) expressing the dsRNA of the gene of interest [102]. These bacterial clones were purchased from Source Bioscience, UK. Their identities were confirmed by sequencing.

### Desiccation Assay

Dauer larvae were washed off the plates and collected in distilled water. Dense suspensions of worms in 5 µl droplets were transferred onto 3.5 cm plastic Petri dishes in duplicates. The dishes were then placed in controlled humidity chambers equilibrated at 98% RH and preconditioned for 4 days [19]. Afterwards, one replicate of each duplicate dish for each strain was transferred into another chamber at 60% RH, whereas the other duplicate was kept in the same 98% RH chamber. After 1 day, all dishes were removed and the worms on the surface of the dishes were rehydrated with distilled water for at least 2 h. Subsequently, worms were transferred to individual 6 cm NGM agar plates seeded with *E. coli* NA22 and kept at 15 °C overnight to recover.

These experiments were repeated on different days for each mutant or RNAi treatment, at least twice. In each experiment, wild type or RNAi non-treated *daf-2* dauer larvae were used as the positive control. The day following rehydration, survivors were counted in the total population and survival rates were calculated as the percentage of worms that survived the whole procedure. On average, 424 ± 363 (median ± median absolute deviation) worms were counted for each replicate. Differences in survival levels at 98% and 60% RH were separately compared to the relevant controls (wild type or *daf-2*) using beta regression [34,103]. The beta distribution of survival rate data was confirmed by one-sample Kolmogorov-Smirnov test. Mean survival rates and their standard errors were also estimated by beta regression. The desiccation sensitivity phenotype of mutants was then categorized into 4 groups: Desiccation tolerant (p ≥ 0.05 compared to the control by beta regression), desiccation sensitive (> 50% survival, p < 0.05), very sensitive (25 - 50% survival, p < 0.05) and extremely sensitive (< 25% survival, p < 0.05).

### Induction of Desiccation-Related Genes and Proteins

Based on our previous results, *daf-2* and wild-type dauer larvae are similar in respect to all parameters that we measured (e.g., morphology, SDS resistance, desiccation tolerance, longevity, etc.) [19,82]. In addition, *daf-2* eggs grown at 25 °C in a liquid culture environment yield a large homogeneous dauer population with no prior desiccation experience. Therefore, for microarray, geLC-MS/MS, and 2D-DIGE analyses of desiccation-induced transcripts and proteins we used *daf-2* dauer larvae grown in liquid culture. These worms, collected in distilled water, were first filtered on 8 µm Isopore TETP membranes (Millipore, USA) and then placed in a desiccation chamber equilibrated at 98% RH [19]. Worms for RNA extraction were kept in this chamber for 24 h to minimize RNA degradation and then collected in distilled water. Worms for protein extraction were kept in the preconditioning chamber for 4 days. Subsequently, they were collected either in SDS lysis buffer (150 mM NaCl, 50 mM Tris-HCl, 1 mM EDTA, 1% SDS (w/v), 0.2% CHAPS (w/v), 0.1% OGP (v/w), 0.7% Triton X-100 (w/v), 250 ng/ml DNase, 250 ng/ml RNase, and 1× protease inhibitor mix (Roche, Germany), pH 7.5) for geLC-MS/MS, or in urea lysis buffer (7 M urea, 2 M thiourea, 30 mM Tris, 4% CHAPS (w/v), and 1× protease inhibitor mix (GE Healthcare, Germany), pH 9.1) for 2D-DIGE. The samples were immediately frozen in liquid nitrogen until RNA and protein extraction.

One of the 4-day desiccated samples removed from the chamber was rehydrated and kept in distilled water for 24 h. These worms were then pelleted and resuspended in urea lysis buffer for subsequent 2D-DIGE analysis.

### RNA and Protein Extraction

Samples for RNA extraction were homogenized by freezing in liquid nitrogen and thawing in a warm water bath without sonication. Total RNA was then extracted using TRIzol Reagent and a PureLink RNA Mini Kit (Invitrogen, Germany). The amount and purity of the RNA were determined by measuring absorbance at 230, 260, and 280 nm using a NanoDrop ND-1000 UV-VIS Spectrophotometer (Thermo Scientific, USA). RNA quality was determined on a 2100 Bioanalyzer (Agilent, Germany) using an RNA 6000 Nano Kit (Agilent, Germany).

Samples for protein extraction were homogenized by freezing in liquid nitrogen and thawing in a water bath with sonication. Samples were thawed in SDS lysis buffer at 60 °C to increase the solubilizing effect of SDS. Homogenates were then centrifuged at 20,000 g to pellet the debris. Subsequently, supernatants were transferred to Nanosep omega membrane centrifugal devices with a molecular weight cutoff of 3 KDa (Pall Corporation, USA) and concentrated by centrifuging at 15,000 g. The amounts of protein in the SDS and urea lysis buffers were quantified using a Micro BCA protein assay kit (Thermo Scientific, USA) and an RC/DC protein measurement kit (Biorad, Germany), respectively.

### Microarray Hybridization and Data Analysis

4×44K oligoarrays (V2: 020186) were purchased from Agilent, Germany. Labeling and hybridization of RNA were performed as recommended by the manufacturer. Four replicates with the highest RNA quality for each of the two conditions (with or without preconditioning) were used. After hybridization, slides were scanned on an Agilent Microarray Scanner C and expression data were generated via Agilent Feature Extraction Software 10.7.1.1.

The default annotation information of Agilent V2: 020186 microarrays was outdated; therefore, it was updated with more recent annotations from WormBase release WS227 by aligning the probe sequences to the *C. elegans* genome and transcriptome. According to this realignment, 20,058 of 20,470 protein-coding genes of *C. elegans* (98%) were targeted by 34,290 probes (1.71 probes/gene).

Probe intensity data were analyzed in R statistical software using the limma package [104] as a part of the BioConductor project [105]. Raw expression data from all chips were imported as median probe intensities and variance-stabilizing normalization was applied [106]. Subsequently, control and deprecated probes were filtered out and the resulting probe-level expression data were summarized into gene-level expression values by Tukey’s median polish algorithm [107,108]. Differential expression of each gene between the two conditions was then calculated using empirical Bayes statistics. Multiple hypothesis testing with the Benjamini-Hochberg correction was applied on calculated p-values [109]. A change in the expression level was considered to be significant if the adjusted p-value was less than 0.001.

The log_2_-scaled FCs (logFCs or differential expression levels) of significantly upregulated and downregulated genes were separately clustered via k-means clustering into four groups for each type of regulation: very low, low, medium, and high (Figure 1A), which were named FCCs. Genes with the lowest logFCs (FCC “very low”) were discarded because their differential expressions were not considered to be biologically significant (Figure 1A). Microarray data are available in the ArrayExpress database (www.ebi.ac.uk/arrayexpress) under accession number E-MEXP-3899.

Functional annotation clustering of the genes that showed significant changes in expression was performed using the DAVID platform v6.7 [31,32]. Functional annotation data from all available Gene Ontology and protein domain databases that are integrated into DAVID were used. Clustering was run with the following parameters: similarity term overlap = 3, similarity threshold = 0.2, initial group membership = 3, final group membership = 3, multiple linkage threshold = 0.5, and EASE = 0.05.

### geLC-MS/MS

Differential expression of genes was investigated at the proteome level by geLC-MS/MS as described previously [33]. Briefly, 50 µg of total protein (as determined using the BCA assay) from biological triplicates of three samples (control, 1 day preconditioned, and 4 days preconditioned) were first separated by one-dimensional SDS-PAGE. Then, each lane was cut into five slices that were independently *in-gel* digested with trypsin, extracted, and vacuum-dried according to a standard protocol [110]. Dried digests were dissolved in 1% formic acid and injected into a Dionex Ultimate 3000 nano HPLC system (Dionex, Germany) directly coupled to an LTQ Orbitrap Velos (Thermo Fisher Scientific, Germany) as described previously [33]. Linear elution gradients of 150 minutes were used. Database searches were performed using Mascot 2.2.04 software (Matrix Science, UK) against the WormBase WS228 protein sequence database. Subsequently, label-free feature extraction and alignment were performed using a customized version of SuperHirn 0.03. Data normalization and peptide-level ANOVA were performed using DanteR 1.0.1.1. False discovery rates were calculated at three stages: At the level of peptide identifications, Mascot’s decoy database option was used with a 5% threshold. At the level of protein identification, the Trans-Proteomic Pipeline’s Protein Prophet tool was used with a probability cutoff of 95%. Finally, to judge the relevance of protein quantifications, the Benjamini-Hochberg FDR method was applied and an adjusted p-value cutoff level of 0.05 was used for significance.

### 2D-DIGE

Fifty micrograms of each protein sample (as determined using RC/DC kit) in urea lysis buffer were labeled with 250 pmol CyDye DIGE Fluor dyes (GE Healthcare, Germany). After labeling, excess dyes were quenched with 10 nmol L-lysine and the samples were reduced in rehydration buffer (7 M urea, 2 M thiourea, 4% CHAPS (w/v), and 50 mM DTT). The samples were pooled and supplemented with ampholytes (BioLytes pH 3–10, BioRad, Germany) in a total volume of 400 µl. This labeled protein mixture was loaded into an immobilized pH gradient strip (linear pH of 3–10) via passive rehydration for 24 h. Following rehydration, isoelectric focusing was performed in a Protean IEF cell (BioRad, Germany) for 55–60 kVh in total. The strip was then equilibrated in equilibration buffer (6 M urea, 2% SDS (w/v), 50 mM Tris, 20% glycerol (v/v), and 130 mM DTT) for 10 min before being placed on a 20 cm wide 12.5% SDS-polyacrylamide gel. Proteins in the strip, along with PageRuler Plus prestained protein weight marker (Fermentas, USA), were separated by SDS-PAGE at 200 V for 6 h. Finally, the gel was scanned using a Typhoon 9410 Variable Mode Imager (GE Healthcare, Germany) at 100 µm/pixel resolution for Cy2 (488 nm excitation, BP 520/40 emission filter), Cy3 (532 nm excitation, BP 580/30 emission filter), and Cy5 (633 nm excitation, BP 670/30 emission filter) at empirically determined photomultiplier tube voltages. After laser scanning, gels were stained with Comassie blue and the spots of interest were cut out. The proteins in these gel slices were extracted and characterized in a similar way as geLC-MS/MS.

### Fatty Acid Analysis by LC-MS

Total lipids were isolated from preconditioned wild-type as well as *fat-1*, *fat-3*, *fat-4*, and *fat-*1,4 mutant dauer larvae via Bligh and Dyer’s method [111]. The organic phase of each sample, normalized by total protein (using BCA assay), was analyzed by reversed-phase liquid chromatography mass spectrometry using an Agilent G1312A pump equipped with an Agilent Autosampler G1329A. Separation employed an Eclipse XDB-C18 column (15 cm × 4.6 mm i.d., 5 µm, Agilent) connected to a Symmetry C18 column (7.5 cm × 4.6 mm i.d., 3.5 µm, Waters) interfaced to a Waters/Micromass LCT TOF mass spectrometer equipped with an ESI. Fatty acid species were separated by isocratic elution using 0.15% ammonium acetate in MeOH/water/MeCN (80:15:5 v/v/v) at 40 °C. The flow rate was set to 1 ml/min with a split to 30 µl/min. The mass spectrometer was operated with a spray voltage of 3 kV and a source temperature of 120 °C. Nitrogen was used as the cone and nebulizing gas. Mass spectra were acquired from the m/z range of 100–1000 controlled with Waters/Micromass MassLynx 4.1 software.

### Bioinformatics Analysis of Uncharacterized Proteins

Protein sequences of uncharacterized proteins were first submitted to conserved domain searches [112] and SMART [113]. For the functional prediction of proteins, domains of unknown function were submitted to HHPRED [114] to detect remote sequence similarity to functionally annotated conserved domains. To integrate the uncharacterized proteins to potential functional networks or pathways, they were submitted to the STRING database [72]. High confidence (0.7) was chosen with all potential protein-protein interaction data sources enabled. The networks were downloaded and imported into Cytoscape [115] for further annotation.

### Sequence Similarity Analysis of IDPs

*Caenorhabditis elegans* DUR-1C (NP_501787.3) protein sequence was compared to *Adineta ricciae* LEA-1A (ABU62809.1), *Adineta vaga* LEA-1B (ADD91471.1), *Arabidopsis lyrata lyrata* LEA (XP_002863597.1), *Brachionus plicatilis* LEA-1 (ADE05593.1), *Caenorhabditis briggsae* LEA-1 (XP_002637990.1), *Caenorhabditis ramenei* DUR-1 (XP_003089862.1), *Caenorhabditis ramenei* LEA-1 (XP_003116339.1), *Caenorhabditis elegans* DUR-1A (NP_501786.2), *Caenorhabditis elegans* LEA-1B (NP_001256170.1), *Medicago truncatula* LEA (XP_003609877.1) and *Oryza sativa* LEA (NP_001049087.1) protein sequences. *Hordeum vulgare* LEA (ABS85196.1) sequence was uaed as the out-group. The tree in Figure S3 was constructed using Geneious software with the following parameters: Blosum62 cost matrix, gap opening penalty = 12, gap extension penalty = 3, global alignment with free end gaps, Jukes-Cantor genetic distance model, and neighbor-joining method.

## Supporting Information

Figure S1 **The concept and experimental design to study the molecular strategies underlying anhydrobiosis.** (A) The concept of anhydrobiosis at the genetic level. DTR mRNAs and proteins (red) can be expressed during dauer formation. Sensing a decrease in ambient humidity (hygrosensation) can activate these mRNAs and proteins, and induce *de* *novo* expression of other DTR mRNAs and proteins (green). This regulation can also occur via post-translational modifications of DTR proteins. Eventually, DTR proteins participate in anhydrobiosis. (B) Experimental design. *daf-2* dauer larvae grown in liquid culture were collected without desiccation, or they were preconditioned at 98% RH for 1 or 4 days, and then collected. One replicate from the 4-day-preconditioned worms was rehydrated for 1 day and then collected. Total RNA was extracted from the non-preconditioned and 1-day-preconditioned samples and used for microarray analyses. Proteins were isolated from all samples and used for geLC-MS/MS or 2D-DIGE analyses. According to the data analysis, candidate genes and pathways were selected and the desiccation tolerances of worms in which these candidates were knocked out or knocked down were tested.
(TIF)

Figure S2 **Comparison of proteomes.** Overlay of false-colored 2D-DIGE images comparing (A) the proteomes of L3 (red) and non-preconditioned dauer (green) larvae, or (B) preconditioned dauer proteomes before (green) and after (red) rehydration. Some proteins that were identified in these gels are annotated with boxes and arrows. (C–E) The regions indicated in rectangles (1–9) are shown in higher magnification for non-preconditioned (–), preconditioned (+), and preconditioned/rehydrated (R) dauer larvae as well as non-preconditioned L3 larvae.
(TIF)

Figure S3 **Similarity of *C. elegans* DUR-1C protein to various DUR and LEA proteins.** *Caenorhabditis elegans* DUR-1C protein sequence was compared to IDP sequences from various organisms. Nematodes, rotifers, and plants are labeled in red, blue, and green, respectively. Scale bar represents a genetic difference of 0.3 substitutions per site.
(TIF)

Figure S4 **Sequence similarity analysis of DUF148 proteins.** (A) Domain structure of DUF148 proteins. All four proteins contain an N-terminal signal peptide followed by a YGG/FGG or LGG-rich region. The DUF148 domain is in the C-terminal half of the proteins. (B) HHPRED finds similarity to the Lipase_chap domain family (PF03280) with more than 95% probability. Secondary structure predictions are shown above and below the family representative, and helical regions are colored in red. Positively charged (orange), negatively charged (yellow), aliphatic (blue), and aromatic (cyan) residues are highlighted.
(TIF)

Figure S5 **STRING interaction network of uncharacterized high FCC proteins.** (A) R05D7.2 interacts with the nucleolar RNA processing machinery. (B) F53A9.2 participates in carbohydrate metabolism. (C) C40H1.3 is associated with a SUMO ligase. (D) C54F6.5 and F41C3.1 are connected to the tumor suppressor and E3 ubiquitin ligase VHL-1 and a cytochrome P450 protein. Queried proteins and their interaction pathways are depicted as red and blue nodes, respectively. Red and cyan lines show evidence of an interaction based on the experimental results, and gray lines indicate interactions identified by text mining. Protein names are colored green or red if their transcripts were upregulated or downregulated during preconditioning, respectively. A high stringency cut-off was used for the STRING algorithm.
(TIF)

Table S1 **Summary of the functional annotation clusters enriched among differentially expressed genes.** Significantly enriched (1.1 to 13.9 fold, p < 0.05, Fisher test) Gene Ontology and protein domain homology terms were clustered in DAVID 6.7 and summarized. See the text and Dataset S2 for details.
(PDF)

Table S2 **Desiccation survival assay results for the mutant screen.** Estimated mean survival rates ± standard errors based on *n* replicates at 98% and 60% RH are presented with the statistical significance (p- values) calculated by beta regression. *daf-2;lea-1*(*RNAi*) and *daf-2;∆∆djr* were compared to *daf-2*, all other mutants are compared to N2. Desiccation sensitivity phenotype is categorized as desiccation tolerant (–), sensitive (+), very sensitive (++) and extremely sensitive (+++). See the text for details.
(PDF)

Table S3 **Prediction of glutathione-S-transferase domains in Cd-responsive proteins.** Pfam sequence search results for the bit scores and e-values of glutathione-S-transferase domains aligned to Cd-responsive proteins at the C- and N-termini are presented.
(PDF)

Table S4 **Sequence similarity search results for uncharacterized high fold change cluster proteins.** For each protein analyzed, information on the identified conserved domains, sequence similarity, and HHPRED analysis results are presented.
(PDF)

Dataset S1 **List of genes that were significantly upregulated and downregulated at the transcriptional level upon preconditioning.** (A) Upregulated genes. (B) Downregulated genes. The column names refer to: ID, the common name of the gene, if applicable, otherwise the systematic name of the gene; Class, gene class; Control and preconditioned, expression level as a measure of the mRNA abundance of the gene before and after preconditioning, respectively; Fold change, differential expression; q-value, Benjamini-Hochberg adjusted p-value of the t-test comparison of mean expression levels for four replicates; FCC, fold change cluster; Description, annotated function of the gene; WormbaseID, the unique WormBase ID code assigned to the gene.
(XLSX)

Dataset S2 **Functional annotation clustering results.** (A) Upregulated and (B) downregulated clusters. The column names refer to: Score, enrichment score; Category, the ontology of functional annotation terms; Count, number of genes annotated with the term; p-value, p-value of the modified Fisher’s exact test; List Total, number of genes in the query list mapped to the ontology; Pop Hits, number of genes with the indicated annotation in the whole genome; Pop Total, number of genes in the whole genome mapped to the ontology; Fold Enrich, fold enrichment of the annotation term in the query list; Genes, Wormbase IDs of the genes enriched in the annotation term.
(XLSX)

Dataset S3 **List of genes that were significantly upregulated and downregulated at the translational level during preconditioning.** The column names refer to: ID, the common name of the gene, if applicable, otherwise the systematic name of the gene; Class, gene class; Fold change, differential expression (for 1 and 4 days of preconditioning); q-value, Benjamini-Hochberg adjusted p-value for the comparison of three replicates (for 1 and 4 days of preconditioning); Description, annotated function of the gene; WormbaseID, the unique WormBase ID code assigned to the gene.
(XLSX)

## References

[1] Crowe JH, Hoekstra FA, Crowe LM (1992) Anhydrobiosis. Annu Rev Physiol 54: 579–599. doi:10.1146/annurev.ph.54.030192.003051. PubMed: 1562184. 1562184

[2] Rascio N, La Rocca N (2005) Resurrection plants: The puzzle of surviving extreme vegetative desiccation. Crit Rev Plant Sci 24: 209–225. doi:10.1080/07352680591002583.

[3] Shannon AJ, Browne JA, Boyd J, Fitzpatrick DA, Burnell AM (2005) The anhydrobiotic potential and molecular phylogenetics of species and strains of Panagrolaimus (Nematoda, Panagrolaimidae). J Exp Biol 208: 2433–2445. doi:10.1242/jeb.01629. PubMed: 15939782. 15939782

[4] Watanabe M (2006) Anhydrobiosis in invertebrates. Applied Entomology and Zoology 41: 15–31. doi:10.1303/aez.2006.15.

[5] Wharton DA, Petrone L, Duncan A, McQuillan AJ (2008) A surface lipid may control the permeability slump associated with entry into anhydrobiosis in the plant parasitic nematode Ditylenchus dipsaci. J Exp Biol 211: 2901–2908. doi:10.1242/jeb.020743. PubMed: 18775927. 18775927

[6] Calahan D, Dunham M, Desevo C, Koshland DE (2011) Genetic analysis of desiccation tolerance in Sachharomyces cerevisiae. Genetics 189: 507–519. doi:10.1534/genetics.111.130369. PubMed: 21840858. 21840858 PMC3189811

[7] Cornette R, Kikawada T (2011) The induction of anhydrobiosis in the sleeping chironomid: Current status of our knowledge. IUBMB Life 63: 419–429. doi:10.1002/iub.463. PubMed: 21547992. 21547992

[8] García AH (2011) Anhydrobiosis in bacteria: from physiology to applications. J Biosci 36: 939–950. doi:10.1007/s12038-011-9107-0. PubMed: 22116292. 22116292

[9] Cano RJ, Borucki MK (1995) Revival and identification of bacterial spores in 25- to 40-million-year-old Dominican amber. Science 268: 1060–1064. doi:10.1126/science.7538699. PubMed: 7538699. 7538699

[10] Shen-Miller J, Mudgett MB, Schopf JW, Clarke S, Berger R (1995) Exceptional seed longevity and robust growth: Ancient Sacred Lotus from China. American Journal of Botany 82: 1367–1380. doi:10.2307/2445863.

[11] Gechev TS, Dinakar C, Benina M, Toneva V, Bartels D (2012) Molecular mechanisms of desiccation tolerance in resurrection plants. Cell Mol Life Sci 69: 3175–3186. doi:10.1007/s00018-012-1088-0. PubMed: 22833170. 22833170 PMC11114980

[12] Martinelli T (2008) In situ localization of glucose and sucrose in dehydrating leaves of Sporobolus stapfianus. J Plant Physiol 165: 580–587. doi:10.1016/j.jplph.2007.01.019. PubMed: 17765358. 17765358

[13] Drennan PM, Smith MT, Goldsworthy D, van Staden J (1993) The occurrence of trehalose in the leaves of the desiccation-tolerant angiosperm Myrothamnus flabellifolius Welw. J Plant Physiol 142: 493–496. doi:10.1016/S0176-1617(11)81257-5.

[14] Farrant JM, Moore JP (2011) Programming desiccation-tolerance: from plants to seeds to resurrection plants. Curr Opin Plant Biol 14: 340–345. doi:10.1016/j.pbi.2011.03.018. PubMed: 21511516. 21511516

[15] Browne JA, Tunnacliffe A, Burnell AM (2002) Anhydrobiosis: plant desiccation gene found in a nematode. Nature 416: 38. doi:10.1038/416038a. PubMed: 11882885. 11882885

[16] França MB, Panek AD, Eleutherio ECA (2007) Oxidative stress and its effects during dehydration. Comp Biochem Physiol B: Biochem Mol Biol 146: 621–631. doi:10.1016/j.cbpa.2006.02.030. PubMed: 16580854. 16580854

[17] Hand SC, Menze MA, Toner M, Boswell L, Moore D (2011) LEA proteins during water stress: Not just for plants anymore. Annu Rev Physiol 73: 115–134. doi:10.1146/annurev-physiol-012110-142203. PubMed: 21034219. 21034219

[18] Gusev O, Cornette R, Kikawada T, Okuda T (2011) Expression of heat shock protein-coding genes associated with anhydrobiosis in an African chironomid Polypedilum vanderplanki. Cell Stress Chaperones 16: 81–90. doi:10.1007/s12192-010-0223-9. PubMed: 20809134. 20809134 PMC3024092

[19] Erkut C, Penkov S, Khesbak H, Vorkel D, Verbavatz J-M et al. (2011) Trehalose renders the dauer larva of Caenorhabditis elegans resistant to extreme desiccation. Curr Biol 21: 1331–1336. doi:10.1016/j.cub.2011.06.064. PubMed: 21782434. 21782434

[20] Erkut C, Penkov S, Fahmy K, Kurzchalia TV (2012) How worms survive desiccation: Trehalose pro water. Worm 1: 61–65. doi:10.1016/j.cub.2011.06.064. PubMed: 24058825. 24058825 PMC3670174

[21] Cassada RC, Russell RL (1975) The dauerlarva, a post-embryonic developmental variant of the nematode Caenorhabditis elegans. Dev Biol 46: 326–342. doi:10.1016/0012-1606(75)90109-8. PubMed: 1183723. 1183723

[22] Tunnacliffe A, Lapinski J, McGee B (2005) A putative LEA protein, but no trehalose, is present in anhydrobiotic bdelloid rotifers. Hydrobiologia 546: 315–321. doi:10.1007/s10750-005-4239-6.

[23] Jönsson KI, Persson O (2010) Trehalose in three species of desiccation tolerant tardigrades. Open Zoology Journal 3: 1–5. doi:10.2174/1874336601003010001.

[24] Sayeed OO, Benzer SS (1996) Behavioral genetics of thermosensation and hygrosensation in Drosophila. Proc Natl Acad Sci U S A 93: 6079–6084. doi:10.1073/pnas.93.12.6079. PubMed: 8650222. 8650222 PMC39192

[25] Liu L, Li Y, Wang R, Yin C, Dong Q et al. (2007) Drosophila hygrosensation requires the TRP channels water witch and nanchung. Nature 450: 294–298. doi:10.1038/nature06223. PubMed: 17994098. 17994098

[26] Merivee E, Must A, Luik A, Williams I (2010) Electrophysiological identification of hygroreceptor neurons from the antennal dome-shaped sensilla in the ground beetle Pterostichus oblongopunctatus. J Insect Physiol 56: 1671–1678. doi:10.1016/j.jinsphys.2010.06.017. PubMed: 20615410. 20615410

[27] Lockhart DJ, Dong H, Byrne MC, Follettie MT, Gallo MV et al. (1996) Expression monitoring by hybridization to high-density oligonucleotide arrays. Nat Biotechnol 14: 1675–1680. doi:10.1038/nbt1296-1675. PubMed: 9634850. 9634850

[28] Cavalcanti ARO, Ferreira R, Gu Z, Li W-H (2003) Patterns of gene duplication in Saccharomyces cerevisiae and Caenorhabditis elegans. J Mol Evol 56: 28–37. doi:10.1007/s00239-002-2377-2. PubMed: 12569420. 12569420

[29] Pellerone FI, Archer SK, Behm CA, Grant WN, Lacey MJ et al. (2003) Trehalose metabolism genes in Caenorhabditis elegans and filarial nematodes. Int J Parasitol 33: 1195–1206. doi:10.1016/S0020-7519(03)00173-5. PubMed: 13678635. 13678635

[30] Kormish JD, McGhee JD (2005) The C. elegans lethal gut-obstructed gob-1 gene is trehalose-6-phosphate phosphatase. Dev Biol 287: 35–47. doi:10.1016/j.ydbio.2005.08.027. PubMed: 16197937. 16197937

[31] Huang DW, Sherman BT, Lempicki RA (2009) Systematic and integrative analysis of large gene lists using DAVID bioinformatics resources. Nat Protoc 4: 44–57. doi:10.1038/nprot.2008.211. PubMed: 19131956. 19131956

[32] Huang DW, Sherman BT, Lempicki RA (2009) Bioinformatics enrichment tools: paths toward the comprehensive functional analysis of large gene lists. Nucleic Acids Res 37: 1–13. doi:10.1093/nar/gkn923. PubMed: 19033363. 19033363 PMC2615629

[33] Vasilj A, Gentzel M, Ueberham E, Gebhardt R, Shevchenko A (2012) Tissue proteomics by one-dimensional gel electrophoresis combined with label-free protein quantification. J Proteome Res 11: 3680–3689. doi:10.1021/pr300147z. PubMed: 22671763. 22671763

[34] Ferrari SLP, Cribari-Neto F (2004) Beta regression for modelling rates and proportions. Journal of Applied Statistics 31: 799–815. doi:10.1080/0266476042000214501.

[35] Hartl FU, Bracher A, Hayer-Hartl M (2011) Molecular chaperones in protein folding and proteostasis. Nature 475: 324–332. doi:10.1038/nature10317. PubMed: 21776078. 21776078

[36] Basha E, O'Neill H, Vierling E (2012) Small heat shock proteins and alpha-crystallins: dynamic proteins with flexible functions. Trends Biochem Sci 37: 106–117. doi:10.1016/j.tibs.2011.11.005. PubMed: 22177323. 22177323 PMC3460807

[37] Coca MA, Almoguera C, Jordano J (1994) Expression of sunflower low-molecular-weight heat-shock proteins during embryogenesis and persistence after germination: localization and possible functional implications. Plant Mol Biol 25: 479–492. doi:10.1007/BF00043876. PubMed: 8049372. 8049372

[38] Schokraie E, Hotz-Wagenblatt A, Warnken U, Frohme M, Dandekar T et al. (2011) Investigating heat shock proteins of tardigrades in active versus anhydrobiotic state using shotgun proteomics. J Zool Syst Evol Res 49: 111–119. doi:10.1111/j.1439-0469.2010.00608.x.

[39] Tyson T, O'Mahony Zamora G, Wong S, Skelton M, Daly B et al. (2012) A molecular analysis of desiccation tolerance mechanisms in the anhydrobiotic nematode Panagrolaimus superbus using expressed sequenced tags. BMC Res Notes 5: 68. doi:10.1186/1756-0500-5-68. PubMed: 22281184. 22281184 PMC3296651

[40] Li D, He X (2009) Desiccation induced structural alterations in a 66-amino acid fragment of an anhydrobiotic nematode late embryogenesis abundant (LEA) protein. Biomacromolecules 10: 1469–1477. doi:10.1021/bm9002688. PubMed: 19408952. 19408952

[41] Liu Y, Chakrabortee S, Li R, Zheng Y, Tunnacliffe A (2011) Both plant and animal LEA proteins act as kinetic stabilisers of polyglutamine-dependent protein aggregation. FEBS Lett 585: 630–634. doi:10.1016/j.febslet.2011.01.020. PubMed: 21251910. 21251910

[42] Chakrabortee S, Tripathi R, Watson M, Kaminski Schierle GS, Kurniawan DP et al. (2012) Intrinsically disordered proteins as molecular shields. Mol Biosyst 8: 210–219. doi:10.1039/c1mb05263b. PubMed: 21909508. 21909508 PMC5365143

[43] Gal TZ, Glazer I, Koltai H (2004) An LEA group 3 family member is involved in survival of C. elegans during exposure to stress. FEBS Lett 577: 21–26. doi:10.1016/j.febslet.2004.09.049. PubMed: 15527756. 15527756

[44] Back P, Matthijssens F, Vlaeminck C, Braeckman BP, Vanfleteren JR (2010) Effects of sod gene overexpression and deletion mutation on the expression profiles of reporter genes of major detoxification pathways in Caenorhabditis elegans. Exp Gerontol 45: 603–610. doi:10.1016/j.exger.2010.01.014. PubMed: 20096764. 20096764

[45] Eisenhaber F, Eisenhaber B, Kubina W, Maurer-Stroh S, Neuberger G et al. (2003) Prediction of lipid posttranslational modifications and localization signals from protein sequences: big-Pi, NMT and PTS1. Nucleic Acids Res 31: 3631–3634. doi:10.1093/nar/gkg537. PubMed: 12824382. 12824382 PMC168944

[46] Thornalley PJ, Langborg A, Minhas HS (1999) Formation of glyoxal, methylglyoxal and 8-deoxyglucosone in the glycation of proteins by glucose. Biochem J 344: 109–116. doi:10.1042/0264-6021:3440109. PubMed: 10548540. 10548540 PMC1220620

[47] Mannervik B (2008) Molecular enzymology of the glyoxalase system. Drug Metabol Drug Interact 23: 13–27. doi:10.1515/DMDI.2008.23.1-2.13. PubMed: 18533362. 18533362

[48] Morcos M, Du X, Pfisterer F, Hutter H, Sayed AAR et al. (2008) Glyoxalase-1 prevents mitochondrial protein modification and enhances lifespan in Caenorhabditis elegans. Aging Cell 7: 260–269. doi:10.1111/j.1474-9726.2008.00371.x. PubMed: 18221415. 18221415

[49] Lee J-Y, Song J, Kwon K, Jang S, Kim C et al. (2012) Human DJ-1 and its homologs are novel glyoxalases. Hum Mol Genet 21: 3215–3225. doi:10.1093/hmg/dds155. PubMed: 22523093. 22523093

[50] Bonifati V, Rizzu P, Squitieri F, Krieger E, Vanacore N et al. (2003) DJ-1 (PARK7), a novel gene for autosomal recessive, early onset parkinsonism. Neurol Sci 24: 159–160. doi:10.1007/s10072-003-0108-0. PubMed: 14598065. 14598065

[51] Liao VH-C, Dong J, Freedman JH (2002) Molecular characterization of a novel, cadmium-inducible gene from the nematode Caenorhabditis elegans. A new gene that contributes to the resistance to cadmium toxicity. J Biol Chem 277: 42049–42059. doi:10.1074/jbc.M206740200. PubMed: 12189149. 12189149

[52] Dong J, Song MO, Freedman JH (2005) Identification and characterization of a family of Caenorhabditis elegans genes that is homologous to the cadmium-responsive gene cdr-1. Biochim Biophys Acta 1727: 16–26. doi:10.1016/j.bbaexp.2004.11.007. PubMed: 15652154. 15652154

[53] Minois N, Carmona-Gutierrez D, Madeo F (2011) Polyamines in aging and disease. Aging (Albany NY) 3: 716–732. 21869457 10.18632/aging.100361 PMC3184975

[54] Macrae M, Plasterk RH, Coffino P (1995) The ornithine decarboxylase gene of Caenorhabditis elegans: cloning, mapping and mutagenesis. Genetics 140: 517–525. PubMed: 7498733. 7498733 10.1093/genetics/140.2.517 PMC1206631

[55] Dufe VT, Lüersen K, Eschbach M-L, Haider N, Karlberg T et al. (2005) Cloning, expression, characterisation and three-dimensional structure determination of Caenorhabditis elegans spermidine synthase. FEBS Lett 579: 6037–6043. doi:10.1016/j.febslet.2005.09.050. PubMed: 16226262. 16226262

[56] Alcázar R, Bitrián M, Bartels D, Koncz C, Altabella T et al. (2011) Polyamine metabolic canalization in response to drought stress in Arabidopsis and the resurrection plant Craterostigma plantagineum. Plant Signal Behav 6: 243–250. doi:10.4161/psb.6.2.14317. PubMed: 21330782. 21330782 PMC3121985

[57] Watts JL (2009) Fat synthesis and adiposity regulation in Caenorhabditis elegans. Trends Endocrinol Metab 20: 58–65. doi:10.1016/j.tem.2008.11.002. PubMed: 19181539. 19181539 PMC2665873

[58] Brock TJ, Browse J, Watts JL (2006) Genetic regulation of unsaturated fatty acid composition in C. elegans. PLoS Genet 2: e108. doi:10.1371/journal.pgen.0020108. PubMed: 16839188. 16839188 PMC1500810

[59] Kniazeva M, Sieber M, McCauley S, Zhang K, Watts JL et al. (2003) Suppression of the ELO-2 FA elongation activity results in alterations of the fatty acid composition and multiple physiological defects, including abnormal ultradian rhythms, in Caenorhabditis elegans. Genetics 163: 159–169. PubMed: 12586704. 12586704 10.1093/genetics/163.1.159 PMC1462428

[60] Watts JL, Browse J (2002) Genetic dissection of polyunsaturated fatty acid synthesis in Caenorhabditis elegans. Proc Natl Acad Sci U S A 99: 5854–5859. doi:10.1073/pnas.092064799. PubMed: 11972048. 11972048 PMC122866

[61] Kahn-Kirby AH, Dantzker JL, Apicella AJ, Schafer WR, Browse J et al. (2004) Specific polyunsaturated fatty acids drive TRPV-dependent sensory signaling in vivo. Cell 119: 889–900. doi:10.1016/j.cell.2004.11.005. PubMed: 15607983. 15607983

[62] Kulas J, Schmidt C, Rothe M, Schunck W-H, Menzel R (2008) Cytochrome P450-dependent metabolism of eicosapentaenoic acid in the nematode Caenorhabditis elegans. Arch Biochem Biophys 472: 65–75. doi:10.1016/j.abb.2008.02.002. PubMed: 18282462. 18282462

[63] Nelson TJT, Cavallaro SS, Yi CLC, McPhie DD, Schreurs BGB et al. (1996) Calexcitin: a signaling protein that binds calcium and GTP, inhibits potassium channels, and enhances membrane excitability. Proc Natl Acad Sci U S A 93: 13808–13813. doi:10.1073/pnas.93.24.13808. PubMed: 8943017. 8943017 PMC19433

[64] Gombos Z, Jeromin A, Mal TK, Chakrabartty A, Ikura M (2001) Calexcitin B is a new member of the sarcoplasmic calcium-binding protein family. J Biol Chem 276: 22529–22536. doi:10.1074/jbc.M010508200. PubMed: 11306567. 11306567

[65] Smith JR, Stanfield GM (2011) TRY-5 is a sperm-activating protease in Caenorhabditis elegans seminal fluid. PLoS Genet 7: e1002375. doi:10.1371/journal.pgen.1002375. PubMed: 22125495. 22125495 PMC3219595

[66] Murphy CT, McCarroll SA, Bargmann CI, Fraser A, Kamath RS et al. (2003) Genes that act downstream of DAF-16 to influence the lifespan of Caenorhabditis elegans. Nature 424: 277–283. doi:10.1038/nature01789. PubMed: 12845331. 12845331

[67] Bock KW (2012) Human UDP-glucuronosyltransferases: Feedback loops between substrates and ligands of their transcription factors. Biochem Pharmacol 84: 1000–1006. doi:10.1016/j.bcp.2012.07.009. PubMed: 22820246. 22820246

[68] Manning G (2005) Genomic overview of protein kinases. WormBook: 1–19. doi:10.1895/wormbook.1.60.1. PubMed: 18050405. PMC4780929 18050405

[69] Sullivan ER, Leahy JG, Colwell RR (1999) Cloning and sequence analysis of the lipase and lipase chaperone-encoding genes from Acinetobacter calcoaceticus RAG-1, and redefinition of a proteobacterial lipase family and an analogous lipase chaperone family. Gene 230: 277–286. doi:10.1016/S0378-1119(99)00026-8. PubMed: 10216267. 10216267

[70] Frenken LG, de Groot A, Tommassen J, Verrips CT (1993) Role of the lipB gene product in the folding of the secreted lipase of Pseudomonas glumae. Mol Microbiol 9: 591–599. doi:10.1111/j.1365-2958.1993.tb01719.x. PubMed: 8412705. 8412705

[71] Brangwynne CP, Eckmann CR, Courson DS, Rybarska A, Hoege C et al. (2009) Germline P granules are liquid droplets that localize by controlled dissolution/condensation. Science 324: 1729–1732. doi:10.1126/science.1172046. PubMed: 19460965. 19460965

[72] Snel B, Lehmann G, Bork P, Huynen MA (2000) STRING: a web-server to retrieve and display the repeatedly occurring neighbourhood of a gene. Nucleic Acids Res 28: 3442–3444. doi:10.1093/nar/28.18.3442. PubMed: 10982861. 10982861 PMC110752

[73] Culotti JG, Russell RL (1978) Osmotic avoidance defective mutants of the nematode Caenorhabditis elegans. Genetics 90: 243–256. PubMed: 730048. 730048 10.1093/genetics/90.2.243 PMC1213887

[74] Hart ACA, Kass JJ, Shapiro JEJ, Kaplan JMJ (1999) Distinct signaling pathways mediate touch and osmosensory responses in a polymodal sensory neuron. J Neurosci 19: 1952–1958. PubMed: 10066248. 10066248 10.1523/JNEUROSCI.19-06-01952.1999 PMC6782580

[75] Solomon AA, Bandhakavi SS, Jabbar SS, Shah RR, Beitel GJG et al. (2004) Caenorhabditis elegans OSR-1 regulates behavioral and physiological responses to hyperosmotic environments. Genetics 167: 161–170. doi:10.1534/genetics.167.1.161. PubMed: 15166144. 15166144 PMC1470864

[76] Wheeler JM, Thomas JH (2006) Identification of a novel gene family involved in osmotic stress response in Caenorhabditis elegans. Genetics 174: 1327–1336. doi:10.1534/genetics.106.059089. PubMed: 16980399. 16980399 PMC1667073

[77] Singh K, Chao MY, Somers GA, Komatsu H, Corkins ME et al. (2011) C. elegans Notch signaling regulates adult chemosensory response and larval molting quiescence. Curr Biol 21: 825–834. doi:10.1016/j.cub.2011.04.010. PubMed: 21549604. 21549604 PMC3100419

[78] Colbert HA, Smith TL, Bargmann CI (1997) OSM-9, a novel protein with structural similarity to channels, is required for olfaction, mechanosensation, and olfactory adaptation in Caenorhabditis elegans. J Neurosci 17: 8259–8269. PubMed: 9334401. 9334401 10.1523/JNEUROSCI.17-21-08259.1997 PMC6573730

[79] Tobin DM, Madsen DM, Kahn-Kirby A, Peckol EL, Moulder G et al. (2002) Combinatorial expression of TRPV channel proteins defines their sensory functions and subcellular localization in C. elegans neurons. Cell 35: 307–318. doi:10.1016/S0896-6273(02)00757-2. 12160748

[80] Albert PS, Brown SJ, Riddle DL (1981) Sensory control of dauer larva formation in Caenorhabditis elegans. J Comp Neurol 198: 435–451. doi:10.1002/cne.901980305. PubMed: 7240452. 7240452

[81] Perens EA, Shaham S (2005) C. elegans daf-6 encodes a patched-related protein required for lumen formation. Dev Cell 8: 893–906. doi:10.1016/j.devcel.2005.03.009. PubMed: 15935778. 15935778

[82] Matyash V, Entchev EV, Mende F, Wilsch-Bräuninger M, Thiele C et al. (2004) Sterol-derived hormone(s) controls entry into diapause in Caenorhabditis elegans by consecutive activation of DAF-12 and DAF-16. PLoS Biol 2: e280. doi:10.1371/journal.pbio.0020280. PubMed: 15383841. 15383841 PMC517820

[83] Battista JR, Park MJ, McLemore AE (2001) Inactivation of two homologues of proteins presumed to be involved in the desiccation tolerance of plants sensitizes Deinococcus radiodurans R1 to desiccation. Cryobiology 43: 133–139. doi:10.1006/cryo.2001.2357. PubMed: 11846468. 11846468

[84] Chakrabortee S, Meersman F, Kaminski Schierle GS, Bertoncini CW, McGee B et al. (2010) Catalytic and chaperone-like functions in an intrinsically disordered protein associated with desiccation tolerance. Proc Natl Acad Sci U S A 107: 16084–16089. doi:10.1073/pnas.1006276107. PubMed: 20805515. 20805515 PMC2941266

[85] de Jesus Pereira E, Panek AD, Eleutherio ECA (2003) Protection against oxidation during dehydration of yeast. Cell Stress Chaperones 8: 120–124. doi:10.1379/1466-1268(2003)008. PubMed: 14627197. 14627197 PMC514863

[86] Roach T, Ivanova M, Beckett RP, Minibayeva FV, Green I et al. (2008) An oxidative burst of superoxide in embryonic axes of recalcitrant sweet chestnut seeds as induced by excision and desiccation. Physiol Plant 133: 131–139. doi:10.1111/j.1399-3054.2007.00986.x. PubMed: 18452494. 18452494

[87] Roach T, Beckett RP, Minibayeva FV, Colville L, Whitaker C et al. (2010) Extracellular superoxide production, viability and redox poise in response to desiccation in recalcitrant Castanea sativa seeds. Plant Cell Environ 33: 59–75. doi:10.1111/j.1365-3040.2009.02053.x. PubMed: 19843255. 19843255

[88] Rizzo AM, Negroni M, Altiero T, Montorfano G, Corsetto P et al. (2010) Antioxidant defences in hydrated and desiccated states of the tardigrade Paramacrobiotus richtersi. Comp Biochem Physiol B Biochem Mol Biol 156: 115–121. doi:10.1016/j.cbpb.2010.02.009. PubMed: 20206711. 20206711

[89] Reardon W, Chakrabortee S, Pereira TC, Tyson T, Banton MC et al. (2010) Expression profiling and cross-species RNA interference (RNAi) of desiccation-induced transcripts in the anhydrobiotic nematode Aphelenchus avenae. BMC Mol Biol 11: 6. doi:10.1186/1471-2199-11-6. PubMed: 20085654. 20085654 PMC2825203

[90] Lee J-Y, Kim C, Kim J, Park C (2013) DJR-1.2 of Caenorhabditis elegans is induced by DAF-16 in the dauer state. Gene 524: 373–376. doi:10.1016/j.gene.2013.04.032. PubMed: 23624124. 23624124

[91] Hayes JD, Flanagan JU, Jowsey IR (2005) Glutathione transferases. Annu Rev Pharmacol Toxicol 45: 51–88. doi:10.1146/annurev.pharmtox.45.120403.095857. PubMed: 15822171. 15822171

[92] Tyson T, Reardon W, Browne JA, Burnell AM (2007) Gene induction by desiccation stress in the entomopathogenic nematode Steinernema carpocapsae reveals parallels with drought tolerance mechanisms in plants. Int J Parasitol 37: 763–776. doi:10.1016/j.ijpara.2006.12.015. PubMed: 17306805. 17306805

[93] Hussain SS, Ali M, Ahmad M, Siddique KHM (2011) Polyamines: Natural and engineered abiotic and biotic stress tolerance in plants. Biotechnol Adv 29: 300–311. doi:10.1016/j.biotechadv.2011.01.003. PubMed: 21241790. 21241790

[94] Houthoofd KK, Braeckman BPB, Lenaerts II, Brys KK, De Vreese AA et al. (2002) Ageing is reversed, and metabolism is reset to young levels in recovering dauer larvae of C. elegans. Exp Gerontol 37: 1015–1021. doi:10.1016/S0531-5565(02)00063-3. PubMed: 12213552. 12213552

[95] Burnell AM, Houthoofd K, O'Hanlon K, Vanfleteren JR (2005) Alternate metabolism during the dauer stage of the nematode Caenorhabditis elegans. Exp Gerontol 40: 850–856. doi:10.1016/j.exger.2005.09.006. PubMed: 16221538. 16221538

[96] McElwee JJ, Schuster E, Blanc E, Thornton J, Gems D (2006) Diapause-associated metabolic traits reiterated in long-lived daf-2 mutants in the nematode Caenorhabditis elegans. Mech Ageing Dev 127: 458–472. doi:10.1016/j.mad.2006.01.006. PubMed: 16522328. 16522328

[97] Clegg JS (1979) Metabolism and the intracellular environment: The vicinal-water network model. In: Drost-Hansen W Clegg JS Cell-Associated Water. London: Cell-associated water. pp. 363–413.

[98] Gadd GM, Chalmers K, Reed RH (1987) The role of trehalose in dehydration resistance of Saccharomyces cerevisiae. FEMS Microbiology Letters 48: 249–254. doi:10.1111/j.1574-6968.1987.tb02551.x.

[99] Welch AZ, Gibney PA, Botstein D, Koshland DE (2013) TOR and RAS pathways regulate desiccation tolerance in Saccharomyces cerevisiae. Mol Biol Cell 24: 115–128. doi:10.1091/mbc.E12-07-0524. PubMed: 23171550. 23171550 PMC3541959

[100] Brenner S (1974) The genetics of Caenorhabditis elegans. Genetics 77: 71–94. PubMed: 4366476. 4366476 10.1093/genetics/77.1.71 PMC1213120

[101] Sulston JE, Brenner S (1974) DNA of Caenorhabditis elegans. Genetics 77: 95–104. PubMed: 4858229. 4858229 10.1093/genetics/77.1.95 PMC1213121

[102] Kamath RS, Martinez-Campos M, Zipperlen P, Fraser AG, Ahringer J (2001) Effectiveness of specific RNA-mediated interference through ingested double-stranded RNA in Caenorhabditis elegans. Genome Biol 2: RESEARCH0002–RESEARCH0002. doi:10.1186/gb-2000-2-1-research0002. PubMed: 11178279. 11178279 PMC17598

[103] Cribari-Neto F, Zeileis A (2010) Beta regression in R. Journal of Statistical Software 34: 1–24.

[104] Smyth GK (2005) Limma: linear models for microarray data. In: Gentleman R Carey V Dudoit S Irizarry R Huber W Bioinformatics and computational biology solutions using R and Bioconductor. New York: Springer pp. 397–420.

[105] Gentleman RC, Carey VJ, Bates DM, Bolstad B, Dettling M et al. (2004) Bioconductor: open software development for computational biology and bioinformatics. Genome Biol 5: R80. doi:10.1186/gb-2004-5-10-r80. PubMed: 15461798. 15461798 PMC545600

[106] Huber W, Heydebreck Von A, Sültmann H, Poustka A, Vingron M (2002) Variance stabilization applied to microarray data calibration and to the quantification of differential expression. Bioinformatics 18: S96–S104. doi:10.1093/bioinformatics/18.suppl_1.S96. PubMed: 12169536. 12169536

[107] Tukey JW (1977) Exploratory data analysis. Reading: Addison-Wesley p. 1.

[108] Irizarry RA, Bolstad BM, Collin F, Cope LM, Hobbs B et al. (2003) Summaries of Affymetrix GeneChip probe level data. Nucleic Acids Res 31: e15–e15. doi:10.1093/nar/gng015. PubMed: 12582260. 12582260 PMC150247

[109] Benjamini Y, Hochberg Y (1995) Controlling the false discovery rate - A practical and powerful approach to multiple testing. J Roy Stat Soc B Met 57: 289–300.

[110] Shevchenko A, Tomas H, Havlis J, Olsen JV, Mann M (2006) In-gel digestion for mass spectrometric characterization of proteins and proteomes. Nat Protoc 1: 2856–2860. doi:10.1038/nprot.2006.468. PubMed: 17406544. 17406544

[111] Bligh E, Dyer W (1959) A rapid method of total lipid extraction and purification. Can J Biochem Physiology 37: 911–917. doi:10.1139/o59-099. 13671378

[112] Marchler-Bauer A, Lu S, Anderson JB, Chitsaz F, Derbyshire MK et al. (2011) CDD: a Conserved Domain Database for the functional annotation of proteins. Nucleic Acids Res 39: D225–D229. doi:10.1093/nar/gkq1189. PubMed: 21109532. 21109532 PMC3013737

[113] Schultz J, Milpetz F, Bork P, Ponting CP (1998) SMART, a simple modular architecture research tool: identification of signaling domains. Proc Natl Acad Sci U S A 95: 5857–5864. doi:10.1073/pnas.95.11.5857. PubMed: 9600884. 9600884 PMC34487

[114] Söding J (2005) Protein homology detection by HMM-HMM comparison. Bioinformatics 21: 951–960. doi:10.1093/bioinformatics/bti125. PubMed: 15531603. 15531603

[115] Smoot ME, Ono K, Ruscheinski J, Wang P-L, Ideker T (2011) Cytoscape 2.8: new features for data integration and network visualization. Bioinformatics 27: 431–432. doi:10.1093/bioinformatics/btq675. PubMed: 21149340. 21149340 PMC3031041

