# Supplementary figures and images for: Molecular Strategies of the Caenorhabditis elegans Dauer Larva to Survive Extreme Desiccation
Source: PLoS One. 2013 Dec 4;8(12):e82473. doi: 10.1371/journal.pone.0082473 (PMC3853187; doi:10.1371/journal.pone.0082473)

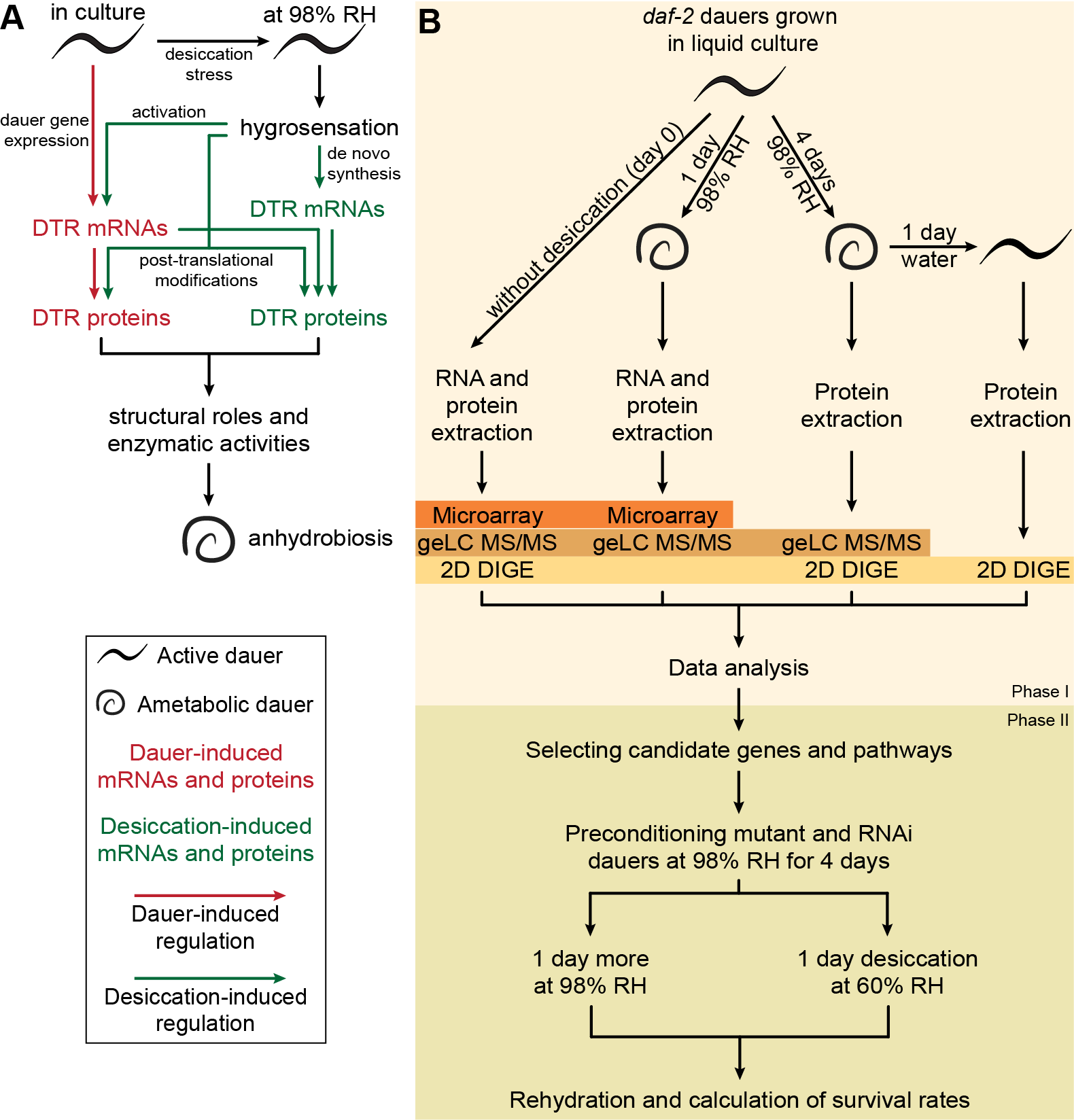

Supplement: Figure S1 — The concept and experimental design to study the molecular strategies underlying anhydrobiosis. (A) The concept of anhydrobiosis at the genetic level. DTR mRNAs and proteins (red) can be expressed during dauer formation. Sensing a decrease in ambient humidity (hygrosensation) can activate these mRNAs and proteins, and induce de novo expression of other DTR mRNAs and proteins (green). This regulation can also occur via post-translational modifications of DTR proteins. Eventually, DTR proteins participate in anhydrobiosis. (B) Experimental design. daf-2 dauer larvae grown in liquid culture were collected without desiccation, or they were preconditioned at 98% RH for 1 or 4 days, and then collected. One replicate from the 4-day-preconditioned worms was rehydrated for 1 day and then collected. Total RNA was extracted from the non-preconditioned and 1-day-preconditioned samples and used for microarray analyses. Proteins were isolated from all samples and used for geLC-MS/MS or 2D-DIGE analyses. According to the data analysis, candidate genes and pathways were selected and the desiccation tolerances of worms in which these candidates were knocked out or knocked down were tested. (TIF) [file pone.0082473.s001.tif]

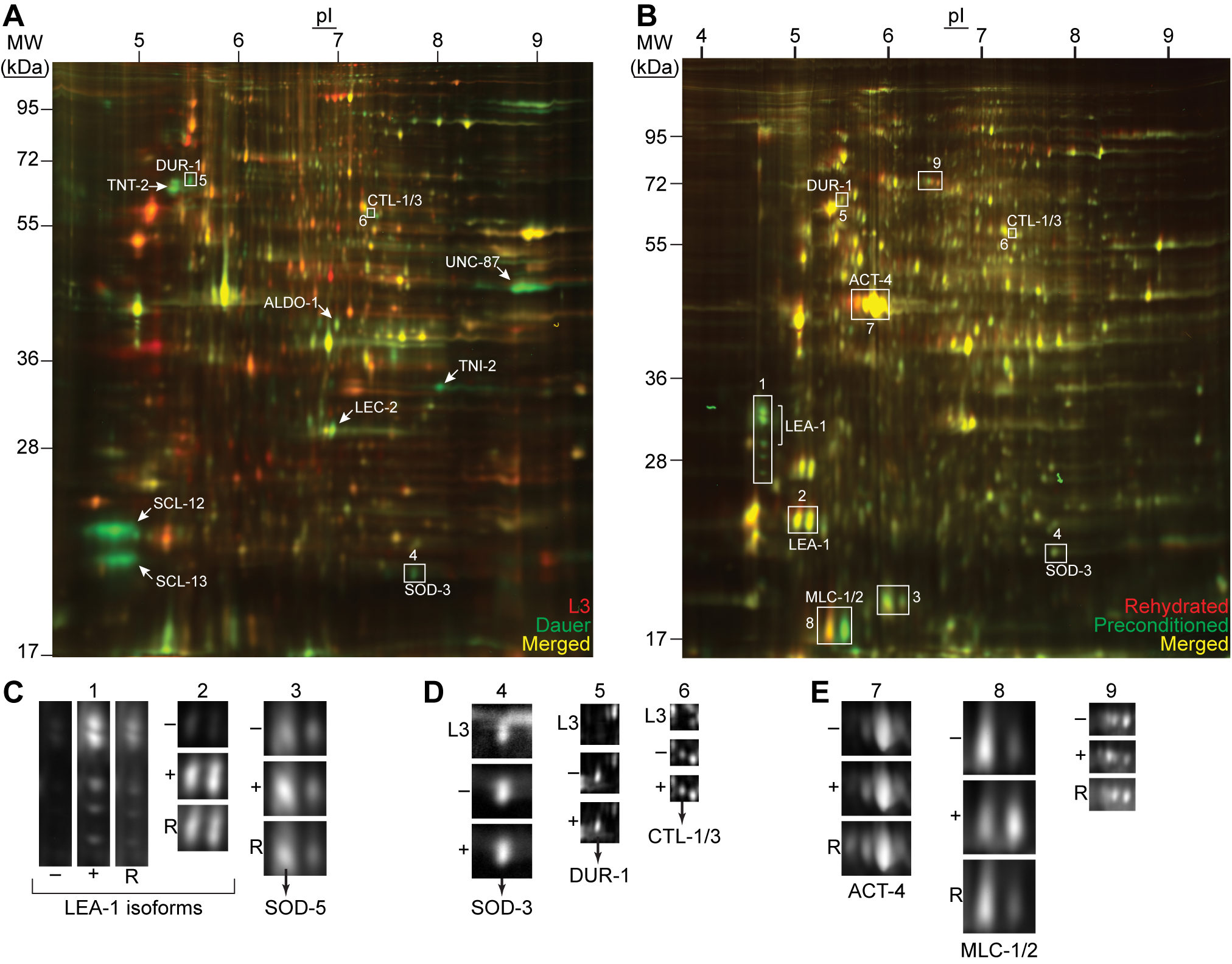

Supplement: Figure S2 — Comparison of proteomes. Overlay of false-colored 2D-DIGE images comparing (A) the proteomes of L3 (red) and non-preconditioned dauer (green) larvae, or (B) preconditioned dauer proteomes before (green) and after (red) rehydration. Some proteins that were identified in these gels are annotated with boxes and arrows. (C–E) The regions indicated in rectangles (1–9) are shown in higher magnification for non-preconditioned (–), preconditioned (+), and preconditioned/rehydrated (R) dauer larvae as well as non-preconditioned L3 larvae. (TIF) [file pone.0082473.s002.tif]

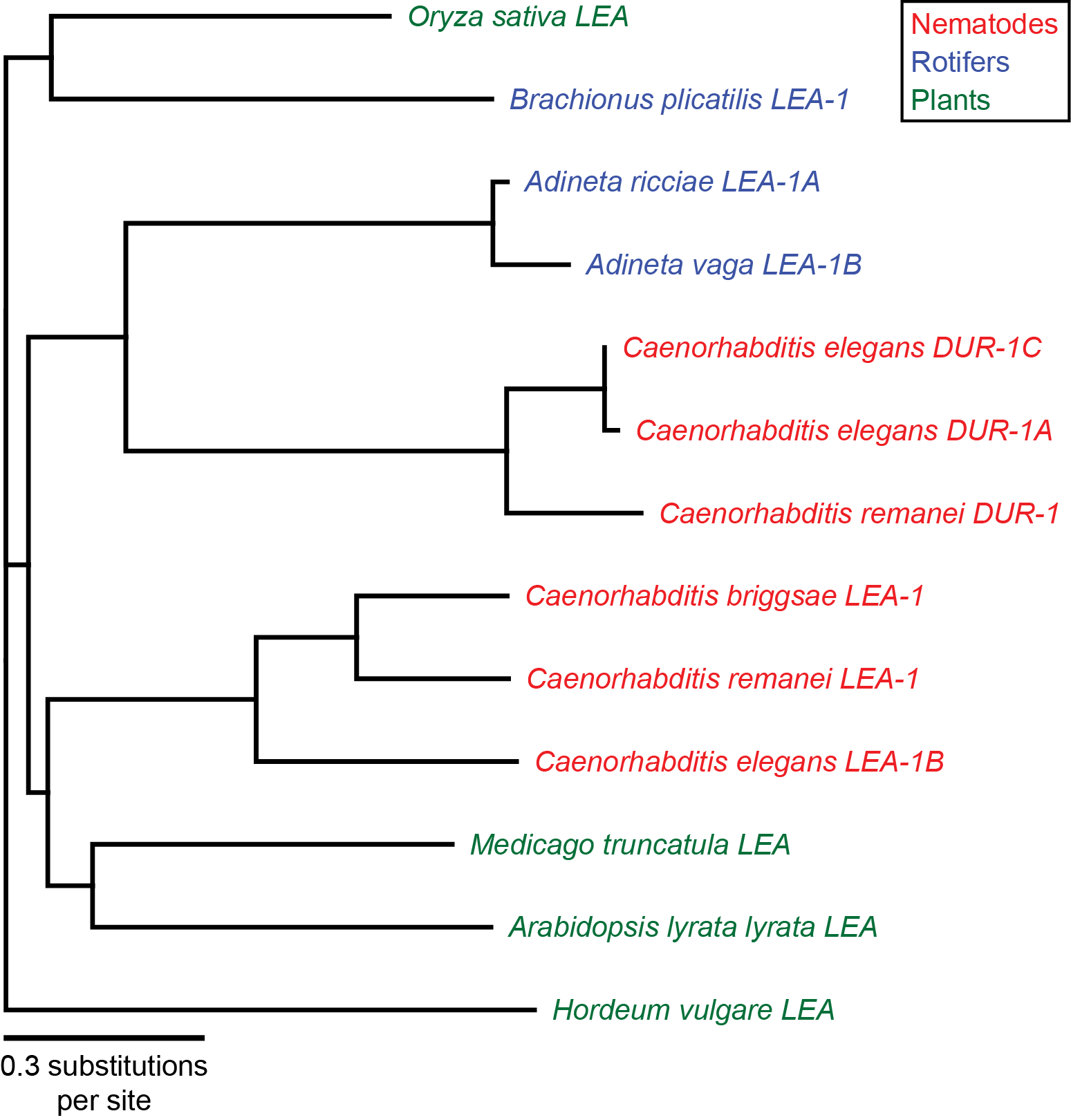

Supplement: Figure S3 — Similarity of C. elegans DUR-1C protein to various DUR and LEA proteins. Caenorhabditis elegans DUR-1C protein sequence was compared to IDP sequences from various organisms. Nematodes, rotifers, and plants are labeled in red, blue, and green, respectively. Scale bar represents a genetic difference of 0.3 substitutions per site. (TIF) [file pone.0082473.s003.tif]

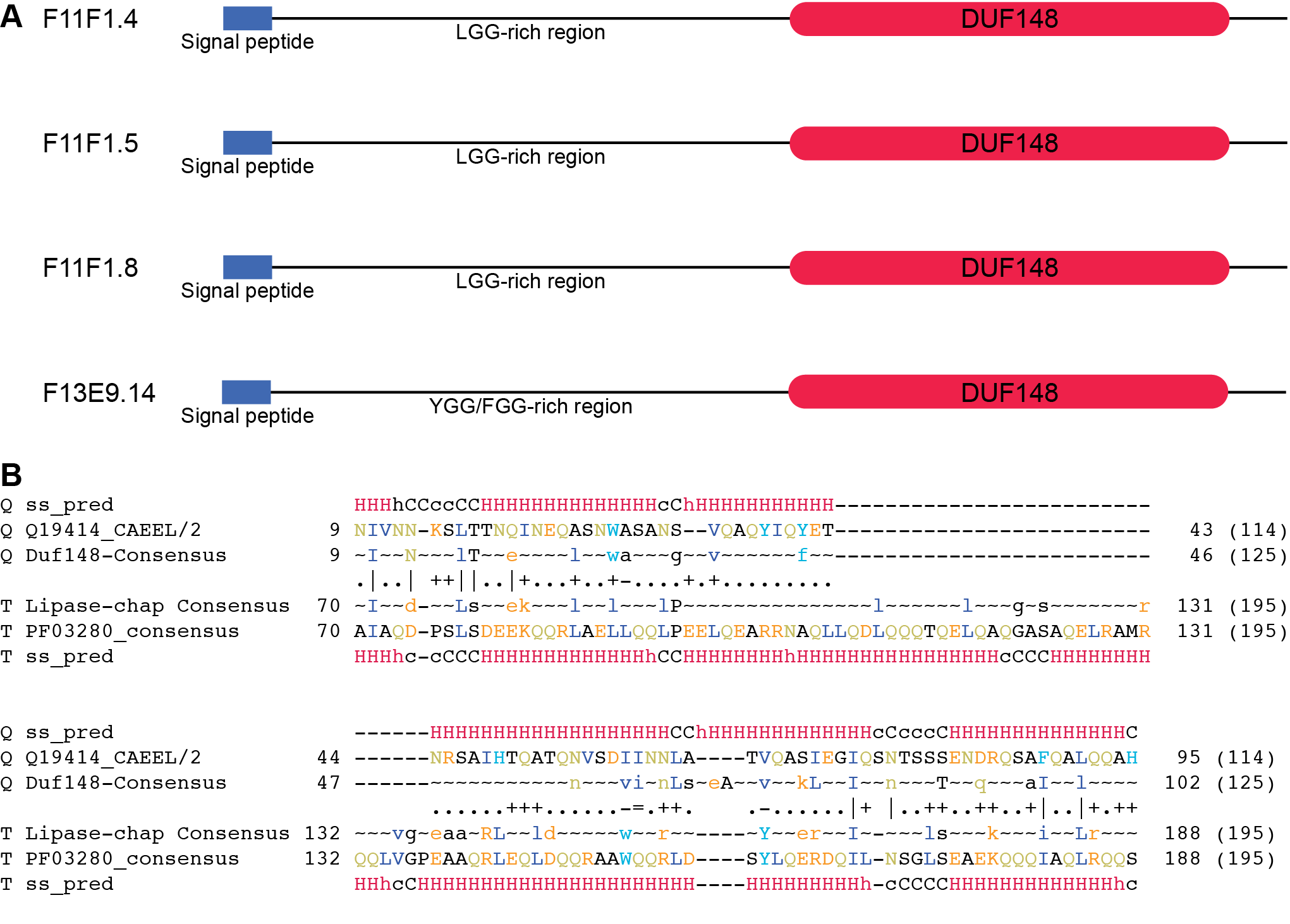

Supplement: Figure S4 — Sequence similarity analysis of DUF148 proteins. (A) Domain structure of DUF148 proteins. All four proteins contain an N-terminal signal peptide followed by a YGG/FGG or LGG-rich region. The DUF148 domain is in the C-terminal half of the proteins. (B) HHPRED finds similarity to the Lipase_chap domain family (PF03280) with more than 95% probability. Secondary structure predictions are shown above and below the family representative, and helical regions are colored in red. Positively charged (orange), negatively charged (yellow), aliphatic (blue), and aromatic (cyan) residues are highlighted. (TIF) [file pone.0082473.s004.tif]

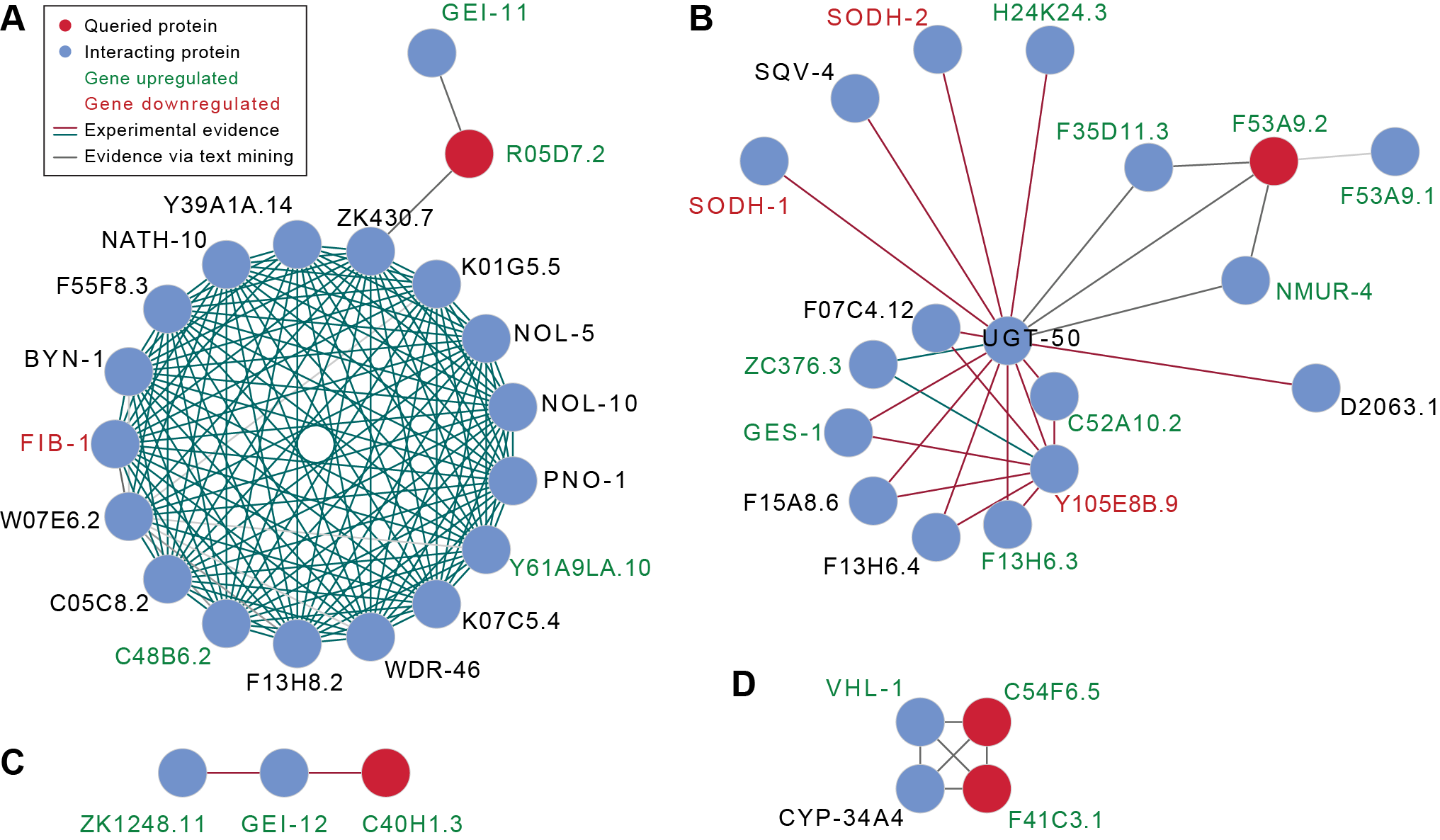

Supplement: Figure S5 — STRING interaction network of uncharacterized high FCC proteins. (A) R05D7.2 interacts with the nucleolar RNA processing machinery. (B) F53A9.2 participates in carbohydrate metabolism. (C) C40H1.3 is associated with a SUMO ligase. (D) C54F6.5 and F41C3.1 are connected to the tumor suppressor and E3 ubiquitin ligase VHL-1 and a cytochrome P450 protein. Queried proteins and their interaction pathways are depicted as red and blue nodes, respectively. Red and cyan lines show evidence of an interaction based on the experimental results, and gray lines indicate interactions identified by text mining. Protein names are colored green or red if their transcripts were upregulated or downregulated during preconditioning, respectively. A high stringency cut-off was used for the STRING algorithm. (TIF) [file pone.0082473.s005.tif]
