# Supplementary material for: Molecular Strategies of the Caenorhabditis elegans Dauer Larva to Survive Extreme Desiccation
Source: PLoS One. 2013 Dec 4;8(12):e82473. doi: 10.1371/journal.pone.0082473 (PMC3853187; doi:10.1371/journal.pone.0082473)
Supplement: Table S1 — Summary of the functional annotation clusters enriched among differentially expressed genes. Significantly enriched (1.1 to 13.9 fold, p < 0.05, Fisher test) Gene Ontology and protein domain homology terms were clustered in DAVID 6.7 and summarized. See the text and Dataset S2 for details. (PDF) [file pone.0082473.s006.pdf]

Table S1. Summary of the functional annotation clusters enriched among differentially expressed genes. Significantly enriched (1.1 to 13.9 fold, $p < 0.05$, Fisher test) Gene Ontology and protein domain homology terms were clustered in DAVID 6.7 and summarized. See the text and Dataset S2 for details.

**Upregulated functional clusters**	**Downregulated functional clusters**
Aldehyde dehydrogenases	Acid phosphatase activity
Alpha/beta hydrolases	Alcohol and short-chain dehydrogenase activity
Cadmium-inducible proteins	Amino acid metabolism and transport
Carboxylesterase and lipase activity	Aspartic, astacin, cysteine, neprilysin, pyroglutamyl, and thrombospondin-like peptidases; carboxypeptidase activity
Carbohydrate and secondary metabolite metabolism	C-type lectins
Chaperonine TCP-1 proteins	Carboxylesterase and (phospho)lipase activity; acyl-CoA thioesterase activity
Chymotrypsin activity	Cuticle development
Cytochrome P450 activity	Domains of unknown function DB, DUF19, DUF23, DUF274, and DUF856
Fatty acid binding activity	Extracellular matrix proteins
Fatty acid desaturation	Lipid glycosylation
Glutathione *S*-transferase activity	Lysozyme activity
Lipid glycosylation	Major sperm protein
Low temperature and salt responsive protein-like	Membrane proteins
Membrane proteins	Neurotransmitter-gated ion-channels and sodium
Mitochondrial substrate/solute carrier activity	Nucleosome assembly
Nuclear hormone receptors	Serpentine type receptors, classes R and X
Oxidoreductase activity	Transthyretin-like proteins
Cysteine-type endopeptidase inhibitors	
Serpentine type receptors, class E	
Short-chain dehydrogenase activity	
Sugar transport	
Phosphate transport	
Superoxide dismutase activity	
Transthyretin-like proteins	
Vitamin B6-binding activity	
