# Supplementary material for: Molecular Strategies of the Caenorhabditis elegans Dauer Larva to Survive Extreme Desiccation
Source: PLoS One. 2013 Dec 4;8(12):e82473. doi: 10.1371/journal.pone.0082473 (PMC3853187; doi:10.1371/journal.pone.0082473)
Supplement: Table S2 — Desiccation survival assay results for the mutant screen. Estimated mean survival rates ± standard errors based on n replicates at 98% and 60% RH are presented with the statistical significance (p- values) calculated by beta regression. daf-2;lea-1(RNAi) and daf-2;∆∆djr were compared to daf-2, all other mutants are compared to N2. Desiccation sensitivity phenotype is categorized as desiccation tolerant (–), sensitive (+), very sensitive (++) and extremely sensitive (+++). See the text for details. (PDF) [file pone.0082473.s007.pdf]

Table S2. Desiccation survival assay results for the mutant screen. Estimated mean survival rates ± standard errors based on *n* replicates at 98% and 60% RH are presented with the statistical significance (p-values) calculated by beta regression. *daf-2;lea-1(RNAi)* and *daf-2;ΔΔdjr* were compared to *daf-2*, all other mutants are compared to N2. Desiccation sensitivity phenotype is categorized as desiccation tolerant (–), sensitive (+), very sensitive (++) and extremely sensitive (+++). See the text for details.

		98% RH			60% RH		
**Strain**	**n**	**Survival**	**p-value**	**Sensitivity**	**Survival**	**p-value**	**Sensitivity**
N2	12	87.5 ± 3.1			82.9 ± 3.6		
daf-2	4	87.8 ± 3.4			91.1 ± 2.6		
**Heat shock proteins**							
F08H9.3	2	62.4 ± 13.4	0.026	+	10.1 ± 6.3	< 0.001	+++
F08H9.4	2	76.2 ± 11.2	0.251	-	52.3 ± 12.9	0.012	+
hsp-12.6	2	87.1 ± 7.8	0.959	-	66.3 ± 12.1	0.135	-
hsp-70	4	81.8 ± 6.9	0.408	-	45.3 ± 9.2	< 0.001	++
**Intrinsically disordered proteins**							
dur-1	5	49.7 ± 8.9	< 0.001	++	12.4 ± 4.6	< 0.001	+++
daf-2;lea-1(RNAi)	2	97.0 ± 2.1	0.039	-	32.9 ± 6.2	< 0.001	++
**Reactive oxygen species defense enzymes**							
sod-1	2	78.3 ± 10.7	0.333	-	53.2 ± 12.9	0.014	+
sod-3	2	80.8 ± 10.0	0.468	-	85.0 ± 8.2	0.814	-
sod-5	3	86.0 ± 6.7	0.832	-	66.0 ± 9.9	0.076	-
gpx-2	3	85.4 ± 6.9	0.767	-	50.2 ± 10.6	0.002	+
gpx-6	2	66.9 ± 12.8	0.058	-	32.8 ± 11.9	< 0.001	++
gpx-7	2	78.9 ± 10.5	0.363	-	15.5 ± 8.4	< 0.001	+++
ctl-1	2	84.4 ± 8.8	0.720	-	42.4 ± 12.8	0.001	++
ctl-3	2	86.6 ± 8.0	0.915	-	89.3 ± 6.6	0.447	-
**Xenobiotic detoxification enzymes**							
daf-2;ΔΔdjr	2	97.9 ± 1.7	0.016	-	79.9 ± 5.3	0.046	+
glod-4	2	78.8 ± 10.6	0.357	-	74.2 ± 10.9	0.407	-
cdr-2	2	62.4 ± 13.4	0.026	+	16.6 ± 8.7	< 0.001	+++
cdr-3	2	80.1 ± 10.2	0.429	-	64.3 ± 12.3	0.099	+
alh-2	2	80.7 ± 10.0	0.460	-	67.0 ± 12.0	0.151	-
**Polyamine biosynthesis enzymes**							
odc-1	2	56.9 ± 13.7	0.009	+	20.5 ± 9.8	< 0.001	+++
spds-1	3	9.7 ± 5.1	< 0.001	+++	5.0 ± 2.9	< 0.001	+++
**Fatty acid modification enzymes**							
fat-5	2	81.9 ± 9.7	0.534	-	9.5 ± 6.0	< 0.001	+++
fat-6	2	48.4 ± 13.9	0.002	++	7.3 ± 4.9	< 0.001	+++
fat-7	2	50.1 ± 13.9	0.002	+	5.3 ± 3.8	< 0.001	+++
fat-3	2	61.2 ± 13.5	0.021	+	10.2 ± 6.3	< 0.001	+++
fat-4	4	27.5 ± 8.5	< 0.001	++	22.5 ± 7.3	< 0.001	+++
fat-1	2	79.2 ± 10.5	0.376	-	64.8 ± 12.2	0.107	-
fat-1,4	2	63.3 ± 13.3	0.031	+	4.3 ± 3.2	< 0.001	+++
cyp-33C9	2	78.6 ± 10.6	0.350	-	57.2 ± 12.8	0.030	+
**Putative hygrosensation proteins**							
daf-6	2	69.8 ± 12.4	0.093	-	28.6 ± 11.4	< 0.001	++
osm-9	2	60.5 ± 13.5	0.018	+	34.9 ± 12.2	< 0.001	++
ocr-1,2,4	3	89.9 ± 5.3	0.700	-	72.8 ± 9.1	0.262	-
osm-11	3	87.3 ± 6.3	0.973	-	43.4 ± 10.5	< 0.001	++
**Novel proteins**							
cex-1	4	69.2 ± 8.9	0.025	+	54.5 ± 9.2	0.002	+
cex-2	4	57.5 ± 9.8	< 0.001	+	16.5 ± 6.1	< 0.001	+++
try-5	4	73.0 ± 8.5	0.064	-	37.6 ± 8.9	< 0.001	++
ugt-1	4	67.6 ± 9.1	0.016	+	31.0 ± 8.4	< 0.001	++
C04G2.2	4	86.3 ± 5.7	0.850	-	51.9 ± 9.2	< 0.001	+
