# Supplementary material for: Molecular Strategies of the Caenorhabditis elegans Dauer Larva to Survive Extreme Desiccation
Source: PLoS One. 2013 Dec 4;8(12):e82473. doi: 10.1371/journal.pone.0082473 (PMC3853187; doi:10.1371/journal.pone.0082473)
Supplement: Table S3 — Prediction of glutathione-S-transferase domains in Cd-responsive proteins. Pfam sequence search results for the bit scores and e-values of glutathione-S-transferase domains aligned to Cd-responsive proteins at the C- and N-termini are presented. (PDF) [file pone.0082473.s008.pdf]

Table S3. Prediction of glutathione-*S*-transferase domains in Cd-responsive proteins. Pfam sequence search results for the bit scores and e-values of glutathione-*S*-transferase domains aligned to Cd-responsive proteins at the C- and N-termini are presented.

		C-terminal		N-terminal	
Protein name	Fold change in transcript	Bit score	E-value	Bit score	E-value
CDR-1	N/S*	42.2	4.8e-11		
CDR-2	6.7	42.2	4.8e-11		
CDR-3	493.0	33.2	3.2e-08		
CDR-4	17.0	29.1	5.9e-07	24.7	1.8e-05
CDR-5	N/S	29.8	3.5e-07		
CDR-6	4.0	26.2	4.9e-06	25.3	1.2e-05
CDR-7	2.2	34.5	1.2e-08	24.0	3.1e-05

* Not significant
