# Supplementary material for: Molecular Strategies of the Caenorhabditis elegans Dauer Larva to Survive Extreme Desiccation
Source: PLoS One. 2013 Dec 4;8(12):e82473. doi: 10.1371/journal.pone.0082473 (PMC3853187; doi:10.1371/journal.pone.0082473)
Supplement: Table S4 — Sequence similarity search results for uncharacterized high fold change cluster proteins. For each protein analyzed, information on the identified conserved domains, sequence similarity, and HHPRED analysis results are presented. (PDF) [file pone.0082473.s009.pdf]

Table S4. Sequence similarity search results for uncharacterized high fold change cluster proteins. For each protein analyzed, information on the identified conserved domains, sequence similarity, and HHPRED analysis results are presented.

Protein	Domain Information	Similarity, HHPRED Results, and Comments
**F11F1.4**	signal peptide; DUF148; finds HisKA by BLAST	HHPRED finds Lipase Chaperone (F03280); Proteobacterial lipase chaperone protein; InterPro: The proteobacterial lipase chaperone is a lipase helper protein that might be involved in the folding of extracellular lipase during its passage through the periplasm; GO: 0051082 unfolded protein binding, 0006457 protein folding, 0016020 membrane; has N-terminal LGG-like repeats; not identical to YGG/FGGs; however, it potentially has the same physical features
**F55C12.6**	DUF1248	GNAT-family acetyltransferase-related (PTHR21471)
**C37A5.6**	SMART finds GRP domain (glycine-rich domain) typically induced by various stresses	Has YGG repeats that are found in PGL-1 in P-granules
**C54F6.15**	Signal peptide	Has four pairs of CxxxC motifs
**F55B12.10**	Has two internal repeats	
**F32F2.2**	Non-coding RNA	
**R01B10.2**	Has two internal repeats	Very acidic protein
**F59A7.11**	Signal peptide	
**C40H1.3**	Has a coiled-coil domain similar to centrosomal protein 104 KDa	Has a highly charged tail; CEP104 is described as a glycine-, glutamate- thienylcyclohexylpiperidine-binding protein
**F13E9.14**	Signal peptide; DUF148	HHPRED finds Lipase Chaperone (F03280); Proteobacterial lipase chaperone protein; InterPro: The proteobacterial lipase chaperone is a lipase helper protein that might be involved in the folding of extracellular lipase during its passage through the periplasm; GO: 0051082 unfolded protein binding, 0006457 protein folding, 0016020 membrane; has additional N-terminally enriched FGG/YGG repeats similar to PGL-1 and C37A5.6; P-granules
**Y67H2A.9**	Annotated as a pseudogene; has two major open reading frames	
**Y6E2A.4**	DUF713; coiled-coil domain	No real homology detected by HHPRED; domain DUF713, potentially involved in worm development and positive regulation of growth
**Y51A2B.8**		No domain found; short, low complexity protein
**R05D7.2**	SPK domain (SM000583); DUF545 in PFAM	SPK is a domain of unknown function that is found in some SET and PHD domain-containing proteins and protein kinases; interacts with T28D9.9; most likely has DNA-binding function or a PP-interacting domain (HHPRED-similarity to Myb-DNA-bind-2 domain)
**C08E8.5**		No domain found; short, low complexity protein
**F14D7.12**		No domain found; short, low complexity protein
**F53A9.2**		Has C-terminal His-stretches; also has two/three GGY-repeats in the N-terminus
**T28D9.9**	Lin-8 domain	Interacts with R05D7.2; LIN-8 domain = Ras-mediated vulval induction antagonist; Lin8 domain (present at sites of transcriptional repressor complexes), interacts with Lin35 Rb. Lin35 Rb is a product of the class B synMuv gene LIN-35, which silences genes required for vulval specification through chromatin modification and remodeling. The biological role of the interaction has not been determined; however, predictions have been made. The interaction shows that class A synMuv genes control vulval induction through transcriptional regulation of gene expression. LIN-8 normally functions as part of a protein complex; however, when the complex is absent, other family members can partially replace LIN-8 activity. HHPRED detects similarity to DNA- and RNA-binding domains; potentially nucleic-acid interacting
**F41C3.1**	DUF1265	No homology to any other domain found by HHPRED
